# Evolution and interpolation of double parton distributions using Chebyshev grids

**DOI:** 10.1140/epjc/s10052-023-11692-8

**Published:** 2023-06-26

**Authors:** Markus Diehl, Riccardo Nagar, Peter Plößl, Frank J. Tackmann

**Affiliations:** 1 Deutsches Elektronen-Synchrotron DESY, Notkestr. 85, 22607 Hamburg, Germany; 2 Università degli Studi di Milano-Bicocca and INFN, Sezione di Milano-Bicocca, Piazza della Scienza 3, 20126 Milan, Italy

## Abstract

Double parton distributions are the nonperturbative ingredients needed for computing double parton scattering processes in hadron–hadron collisions. They describe a variety of correlations between two partons in a hadron and depend on a large number of variables, including two independent renormalization scales. This makes it challenging to compute their scale evolution with satisfactory numerical accuracy while keeping computational costs at a manageable level. We show that this problem can be solved using interpolation on Chebyshev grids, extending the methods we previously developed for ordinary single-parton distributions. Using an implementation of these methods in the C++ library ChiliPDF, we study for the first time the evolution of double parton distributions beyond leading order in perturbation theory.

## Introduction

Double parton scattering (DPS) is a mechanism in hadron–hadron collisions in which two partons from each hadron initiate two separate hard-scattering processes. The Tevatron and the LHC have provided evidence for this mechanism for a wide range of final states with heavy flavors, jets, photons, or electroweak gauge bosons [1–6]. While often suppressed compared with single-parton scattering, DPS can be significant and even dominate in specific final states or kinematic regions. A prominent example is like-sign *W* production [5, 7–13], which (via its leptonic decay) also provides a background to searches for physics beyond the Standard Model [14, 15]. Significant progress in the theory description of DPS has been made in the last decade [16–24], and there is a sustained interest in phenomenological aspects (see for instance the recent study in [25] and the references therein). A detailed account of theoretical and experimental aspects of DPS is given in Ref. [26].

Double parton distributions (DPDs) are the nonperturbative quantities needed to compute DPS cross sections. They quantify a variety of correlations between two partons in the proton [19, 20, 27] and thus reveal aspects of hadron structure that are not accessible in ordinary parton distributions (PDFs). Our knowledge of DPDs is still quite limited, although there is considerable activity in devising theory guided ansätze [28–31] and in computing DPDs using lattice QCD [32] or quark models [33–40].

Reflecting the multitude of information they contain, DPDs depend on a large number of variables, namely the momentum fractions $x_1$ and $x_2$ of the two partons, the transverse distance *y* between them, and the renormalization scales $\mu _1$ and $\mu _2$ associated with each parton (corresponding to the factorization scales in the two hard-scattering processes that the partons initiate). Their scale dependence is well understood (see Eq. (1) below), but the numerical delivery of evolved DPDs is a computational challenge. We believe that this represents one of the bottlenecks for using realistic forms of DPDs in predictions, either at the analytical level or in the form of Monte Carlo event generators [41–45]. In the present work, we present a method that addresses this challenge, allowing for high numerical accuracy at a moderate computational cost.

We recall that the evolution equations for PDFs can be solved numerically either by discretization in the momentum fraction *x* or in Mellin space, and that public codes are available for both options, see for instance Refs. [46–50] and [51–53]. For DPDs, even the simplest realistic initial conditions for evolution have a correlated dependence on $x_1$ and $x_2$ (see Eqs. (7) and (8) below), which precludes the analytic computation of complex Mellin moments in these two variables. As far as we can see, this makes a discretization in $x_1$ and $x_2$ inevitable.

The main reason why the handling of evolved distributions is much more demanding for DPDs than for PDFs is the sheer amount of computer memory needed to store the former. As an example, consider interpolation grids for $x_1$ and $x_2$ with 64 points (for PDFs, this is at the lower end of typical grid sizes in the LHAPDF interface [54]), and grids with 48 points for *y*, $\mu _1$, and $\mu _2$. This corresponds to $64^{2} \times 48^3 \approx 4.5 \times 10^8$ real numbers and thus (with 8 bytes for a double precision floating-point number) to $3.4 {\text {GiB}}$ per parton flavor combination. A full unpolarized DPD with 5 active quark flavors has $11^2 = 121$ parton flavor combinations. Getting evolved DPDs from pre-computed grids would thus require an enormous amount of memory (with an added penalty of accessing this memory for interpolation in the five variables). The path we have chosen instead is to store a DPD only for a pair of initial scales, and to evolve “on the fly” to the desired values of $\mu _1$ and $\mu _2$. In the above example, this reduces the memory imprint to $64^2 \times 48$ points and thus to $1.5 {\text {MiB}}$ per flavor combination.

We will see in Sect. 3.4 that the computational effort for evolving a DPD with $p_x$ grid points in $x_1$ and $x_2$ scales like $p_x^3$, whilst the scaling with the number $p_y$ of points in *y* is at most linear. It is therefore of great importance to limit the size of interpolation grids for the momentum fractions, without sacrificing numerical accuracy. In our previous work [55] we found that a highly accurate interpolation and evolution of PDFs in *x* space is possible using Chebyshev grids. In the present paper, we show how to adapt this approach to the case of DPDs. We find that with $p_x \sim 54$ and $p_y \sim 40$ to 56 points, one can achieve high accuracy for the interpolation and integration of DPDs with $x_1, x_2 \ge 10^{-5}$ and $y \ge (7 \,{\textrm{TeV}})^{-1}$.

We have implemented our approach in the C++ library ChiliPDF,^1^ which is under development and which will be made public in the future. Our implementation demonstrates that the methods we will describe do work in practice. It also allows us to explore a number of physics aspects. So far, DPD evolution has been studied only with DGLAP kernels at leading order (LO). We extend this to next-to-leading and next-to-next-to-leading order (NLO and NNLO). Moreover, our implementation includes the change in the number of active quark flavors at specified matching scales, and access to distributions with different scales ($\mu _1$ and $\mu _2$) and flavor numbers ($n_{1}$ and $n_{2}$) for the two partons. Last but not least, we can evolve DPDs for polarized partons, which may for instance have measurable impact on observables in like-sign *W* production according to the studies in Refs. [12, 13].

This paper is organized as follows. In Sect. 2.1 we recall some basics about DPDs and set up the corresponding notation. Using our implementation for a physics study, we compare in Sect. 2.2 the evolution of DPDs at LO, NLO, and NNLO. In Sect. 3, we summarize the method we developed in Ref. [55] for interpolating and evolving PDFs and describe its extension to DPDs. We study the joint interpolation in the two momentum fractions $x_1$ and $x_2$ in Sect. 4, and the evolution in the two scales $\mu _1$ and $\mu _2$ in Sect. 5. Section 6 is devoted to the interpolation in the distance *y* between the partons, from some minimum value to infinity. In Sect. 7 we cross check our implementation against the DPD evolution code DOVE, which was originally introduced in Ref. [28] and further developed in subsequent work. We conclude in Sect. 8. In Appendix A we prove an important statement about the independence of DPD evolution and flavor matching on the path taken in the $(\mu _1, \mu _2)$ plane.

## Basics and main results

### Theory framework

To begin with, let us recall some properties of DPDs that are relevant to this work. A DPD $F^{n_{1}, n_{2}}_{a_1 a_2}(x_1, x_2, y; \mu _1, \mu _2)$ depends on the longitudinal momentum fractions $x_1$ and $x_2$ of the two partons, on their distance *y* in the transverse plane, and on the factorization scales $\mu _1$ and $\mu _2$ associated with each parton. The labels $a_1$ and $a_2$ specify the flavor and polarization of each parton. DPDs involving transverse quark or linear gluon polarization have open Lorenz indices and depend on the difference ${\vec {y}}$ of the transverse parton positions rather than on the length *y* of this vector. As specified in section 2 of Ref. [56], they can be decomposed into scalar distributions that depend on *y*. The evolution and matching equations discussed below hold separately for these scalar distributions. Throughout this work, we consider only DPDs in the color-singlet channel, i.e. where the color is summed over separately for each of the two extracted partons.

Notice that we allow different numbers $n_{1}$ and $n_{2}$ of active quark flavors for the two partons, which is useful when the hard-scattering processes initiated by $a_1$ and $a_2$ take place at very different scales. Technically, $n_{1}$ and $n_{2}$ are specified by the renormalisation prescription for the twist-two operator pertaining to parton $a_1$ and $a_2$, respectively.

The scale dependence of a DPD is given by the evolution equations

1 $$\begin{aligned}&\frac{\textrm{d}}{\textrm{d}\ln \mu _1^2} \, F^{n_{1}, n_{2}}_{a_1 a_2}(x_1,x_2,y; \mu _1, \mu _2)\nonumber \\&\quad = \sum _{b_1} \Bigl [ P_{a_1 b_1}^{n_{1}}(\mu _1) \underset{1}{\otimes } F^{n_{1}, n_{2}}_{b_1 a_2}(x_2, y; \mu _1, \mu _2) \Bigr ](x_1) , \nonumber \\&\frac{\textrm{d}}{\textrm{d}\ln \mu _2^2} \, F^{n_{1}, n_{2}}_{a_1 a_2}(x_1,x_2,y; \mu _1, \mu _2)\nonumber \\&\quad = \sum _{b_2} \Bigr [ P_{a_2 b_2}^{n_{2}}(\mu _2) \underset{2}{\otimes } F^{n_{1}, n_{2}}_{a_1 b_2}(x_1, y; \mu _1, \mu _2) \Bigr ](x_2) \end{aligned}$$

with separate Mellin convolutions

2 $$\begin{aligned}&\Bigr [ P(\mu _1) \underset{1}{\otimes } F(x_2, y; \mu _1, \mu _2) \Bigr ](x_1) \nonumber \\&\quad = \int _{x_1}^{1} \frac{\textrm{d}z}{z}\; P(z; \mu _1)\, F\Bigl ( \frac{x_1}{z}, x_2, y; \mu _1, \mu _2 \Bigr ) , \nonumber \\&\Bigr [ P(\mu _2) \underset{2}{\otimes } F(x_1, y; \mu _1, \mu _2) \Bigr ](x_2) \nonumber \\&\quad = \int _{x_2}^{1} \frac{\textrm{d}z}{z}\; P(z; \mu _2)\, F\Bigl ( x_1, \frac{x_2}{z}, y; \mu _1, \mu _2 \Bigr ) \end{aligned}$$

for the two momentum fractions. Although we define each convolution integral in Eq. (2) with the upper integration limit equal to 1, the effective integration range is reduced by the support property

3 $$\begin{aligned} F^{n_{1}, n_{2}}_{a_1 a_2}(x_1,x_2,y; \mu _1, \mu _2) = 0 \quad \text {for } x_1 + x_2 > 1 \end{aligned}$$

of DPDs, which is conserved by the evolution equations (1). The evolution kernels $P^{n_{}}_{a b}(z; \mu )$ in Eq. (1) are identical to the familiar DGLAP kernels for the evolution of PDFs with $n_{}$ active quark flavors. Note also that the distance *y* plays no active role in the scale evolution, so that each “slice” of a DPD at fixed *y* evolves by itself.

We remark in passing that the homogeneous evolution equations (1) hold for DPDs depending on the interparton distance *y*. An inhomogeneous term appears if one integrates the distributions over ${\vec {y}}$ or performs a Fourier transform from ${\vec {y}}$ to its conjugate transverse momentum [19, 24]. This term is closely related with the splitting contribution (7) discussed below and has been extensively studied in the earlier literature [28, 57–60]. Throughout this work, we will exclusively work with DPDs that depend on *y* and evolve as given in Eq. (1).

The transition from $n_{} - 1$ to $n_{}$ active flavors in a DPD is described by matching equations

4 $$\begin{aligned}&F_{a_1 a_2}^{n_{1}, n_{2}}(x_1,x_2,y; \mu _1, \mu _2) = \sum _{b_1} \Bigl [ A_{a_1 b_1}^{n_{1}}(m_{n_{1}}; \mu _1)\nonumber \\&\quad \underset{1}{\otimes } F_{b_1 a_2}^{n_{1} - 1, n_{2}}(x_2, y;\mu _1, \mu _2) \Bigr ](x_1) , \nonumber \\&F_{a_1 a_2}^{n_{1}, n_{2}}(x_1,x_2,y; \mu _1, \mu _2) = \sum _{b_2} \Bigl [ A_{a_2 b_2}^{n_{2}}(m_{n_{2}}; \mu _2) \nonumber \\&\quad \underset{2}{\otimes } F_{a_1 b_2}^{n_{1}, n_{2} - 1}(x_1, y;\mu _1, \mu _2) \Bigr ](x_2) , \end{aligned}$$

where $m_{n_{}}$ is the mass of the quark with flavor number $n_{}$ and the matching kernels $A^{n_{}}_{a b}(z, m_{n_{}}; \mu )$ are the same as the ones for PDFs [61]:

5 $$\begin{aligned} f_{a}^{n_{}}(x; \mu ) = \sum _{b} \Bigl [ A_{a b}^{n_{}}(m_{n_{}}; \mu ) \underset{}{\otimes } f_{b}^{n_{} - 1}(\mu ) \Bigr ](x). \end{aligned}$$

Equation (1) is a coupled set of differential equations in $\mu _1$ and $\mu _2$. In Appendix A we show that with an initial condition at some point $(\mu _{0 1}, \mu _{0 2})$ one obtains a unique DPD regardless of the evolution path taken in the $(\mu _1, \mu _2)$ plane. We will also show that the result of successive flavor matching does not depend on the order in which the flavor thresholds for the two partons are crossed. Note that the path independence of evolution and flavor matching in the $(\mu _1, \mu _2)$ plane is exact even if the perturbative expansions of the DGLAP and matching kernels are truncated. This is in contrast to the independence on the scale at which the flavor matching is performed, which only holds to the perturbative order of the truncation.

The following evolution and matching kernels are currently implemented in ChiliPDF and used in the present work:

- DGLAP evolution for unpolarized and longitudinally polarized partons up to NNLO (order $\alpha _s^3$). For the NNLO kernels we use the approximate parameterized forms given in Refs. [62–65]. The kernels in these references were confirmed by the calculations in Refs. [66, 67].
- DGLAP evolution up to NLO for transversely polarized quarks and linearly polarized gluons, with the kernels given in Refs. [68, 69].
- Flavor matching for unpolarized partons up to NNLO, i.e. order $\alpha _s^2$. The corresponding kernels were computed independently in Refs. [61, 70–72], and we verified that they agree with each other.
- Flavor matching for polarized partons up to NLO. At order $\alpha _s$, the matching kernels are directly proportional to the DGLAP kernels for the same polarization.

We now take a closer look at the dependence of DPDs on the kinematic variables $x_1, x_2$, and *y*. The *y* dependence is not directly observable, because DPDs enter cross sections (differential or integrated) via so-called double parton luminosities

6 $$\begin{aligned}&{\mathcal {L}}_{a_1 a_2, b_1 b_2}^{n_{1}, n_{2}}(x_1, x_2, {\bar{x}}_1, {\bar{x}}_2; \mu _1, \mu _2, y_{\min }) \nonumber \\&\quad = \int \textrm{d}^2 {\vec {y}} \;\, \theta (y - y_{\min }) \; F_{a_1 a_2}^{n_{1}, n_{2}}(x_1,x_2,y; \mu _1, \mu _2) \, \nonumber \\&\qquad \times F_{b_1 b_2}^{n_{1}, n_{2}}({\bar{x}}_1,{\bar{x}}_2,y; \mu _1, \mu _2) , \end{aligned}$$

where *y* is integrated over with a lower cutoff $y_{\min }$ of order $1 / \min (\mu _1, \mu _2)$. The origin of this cutoff is explained in Ref. [24], where it is also shown how the dependence on this cutoff is removed when the cross sections for single and double parton scattering are combined.

The form in Eq. (6) holds for unpolarized and longitudinally polarized partons; for transverse quark or linear gluon distributions additional tensors depending on ${\vec {y}}$ appear under the integral.

At sufficiently small distances *y*, the dominant contribution to a DPD arises from the perturbative splitting of a single parton into the two observed partons $a_1$ and $a_2$. At leading order in the coupling, this mechanism gives [19, 24]

7 $$\begin{aligned}&F_{a_1 a_2}^{\text {spl}}(x_1,x_2, y; \mu _{y}, \mu _{y}) = \frac{1}{\pi y^2}\, \frac{\alpha _s(\mu _{y})}{2\pi }\nonumber \\&\quad \times V^{(1)}_{a_1 a_2, a_0} \biggl ( \frac{x_1}{x_1 + x_2} \biggr )\, \frac{f_{a_0}(x_1 + x_2;\mu _{y})}{x_1 + x_2} , \end{aligned}$$

where the scale $\mu _{y}$ should be taken of order 1/*y* so as to avoid large logarithms $\ln (y \mu )$ in higher-order corrections. In addition, there is an “intrinsic” two-parton contribution, which lacks the $1/y^2$ enhancement of the splitting part at small *y* but grows more strongly at small $x_1$ and $x_2$ for gluons and sea quarks.

At distances *y* of nonperturbative size, our knowledge of DPDs is rather limited, and in practice one needs to make a model ansatz. Where needed, we will take recourse to the model used in Refs. [24, 30], where a DPD is written as a sum $F^{\text {int}} + F^{\text {spl}}$ of intrinsic and splitting parts at all *y*. At a starting scale $\mu _0$, the intrinsic part of the DPD is assumed to be proportional to the product of two PDFs:

8 $$\begin{aligned}&F_{a_1 a_2}^{\text {int}}(x_1,x_2, y; \mu _0, \mu _0) = \frac{1}{4\pi h_{a_1 a_2}}\, \exp \biggl [ -\frac{y^2}{4h_{a_1 a_2}} \biggr ]\, \nonumber \\&\quad \times \rho (x_1, x_2)\, f_{a_1}(x_1;\mu _0)\, f_{a_2}(x_2;\mu _0), \end{aligned}$$

with a phase space factor

9 $$\begin{aligned} \rho (x_1, x_2)=\frac{(1 - x_1 - x_2)^r}{(1 - x_1)^r (1 - x_2)^r} \end{aligned}$$

to ensure that the DPD smoothly goes to zero at the kinematic boundary $x_1 + x_2 \rightarrow 1$. Other models in the literature take only a power $(1-x_1-x_2)^r$; for the motivation of the form (9) we point to section 3.2 in Ref. [28].

The splitting term in the model of Refs. [24, 30] is given by the perturbative splitting expression (7) multiplied with the same exponential factor $\exp \bigl [ - y^2 / (4 h_{a_1 a_2}) \bigr ]$ as in the intrinsic part. This retains the correct small-*y* limit whilst providing a more realistic decrease at large *y*.

### Quantitative impact of higher orders in DPD evolution

With the methods presented in this work, we are able to evolve DPDs at different perturbative orders, for all relevant polarization combinations and with different scales and active flavor numbers of the two partons. As a demonstration, we now compare DPDs evolved at LO, NLO, and NNLO from a common starting condition.

**Fig. 1:**
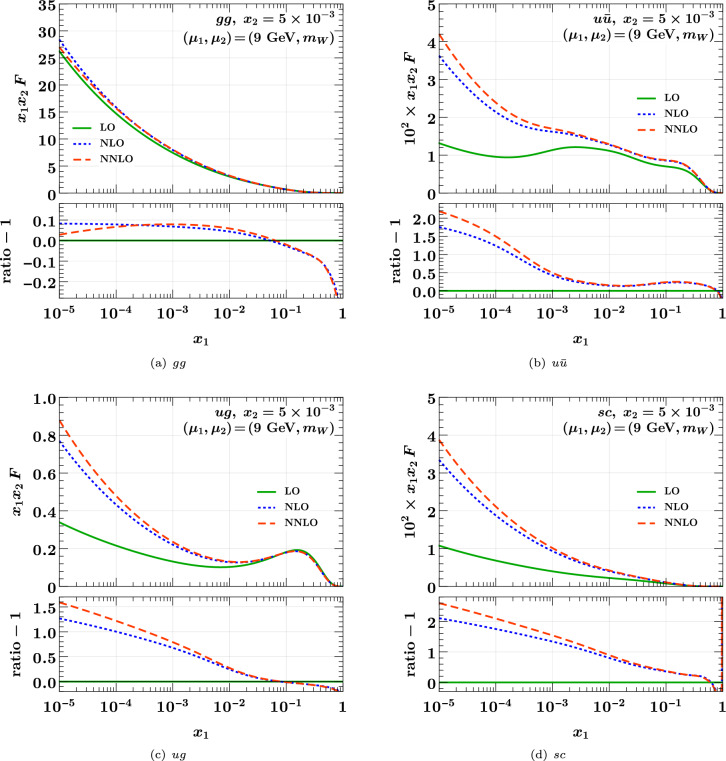
Unpolarized DPDs at $x_2 = 5 \times 10^{-3}$, evolved from $(\mu _1, \mu _2) = (2 \,{\textrm{GeV}}, 2 \,{\textrm{GeV}})$ to $(9 \,{\textrm{GeV}}, m_W)$ at different perturbative orders. The starting conditions and the procedure of flavor matching are specified in the text. The ratio shown in the small panels is taken with respect to the LO result

As starting condition we take the perturbative splitting form (7) with $n_{1} = n_{2} = 3$ active flavors. The splitting formula is evaluated at $\mu _y = 2 \,{\textrm{GeV}}$ and $y \approx 0.561 \,{\textrm{GeV}}^{-1}$, which corresponds to the scale choice $\mu _y = b_0 / y$ with $b_0 = 2 e^{-\gamma _E} \approx 1.12$, where $\gamma _E$ is the Euler–Mascheroni constant. This choice is suggested by the form of the NLO corrections to the splitting formula (7) and was found to keep these corrections at a moderate size [73, 74]. For the PDFs in the DPD splitting formula, we take the default NNLO set of the MSHT20 parameterization [75].^2^ The parameterization assumes quark masses $m_c = 1.4 \,{\textrm{GeV}}$, $m_b = 4.75 \,{\textrm{GeV}}$ and a five-flavor coupling $\alpha _s(m_Z) = 0.118$.

We evolve these initial conditions to $\mu _1 = 9 \,{\textrm{GeV}}$ with $n_{1} = 3$ for the first parton and to $\mu _2 = m_W$ with $n_{2} = 5$ for the second one. This is a setting relevant for the production of a $J/\hspace{-0.83328pt}\Psi$ and a *W*, a channel for which double parton scattering has in fact been observed experimentally [4, 76]. Flavor matching for the second parton is performed at the charm and bottom quark masses, with kernels of the same order (LO, NLO, or NNLO) as the evolution kernels. An exception is longitudinally polarized evolution at NNLO, where we use NLO flavor matching.

**Fig. 2:**
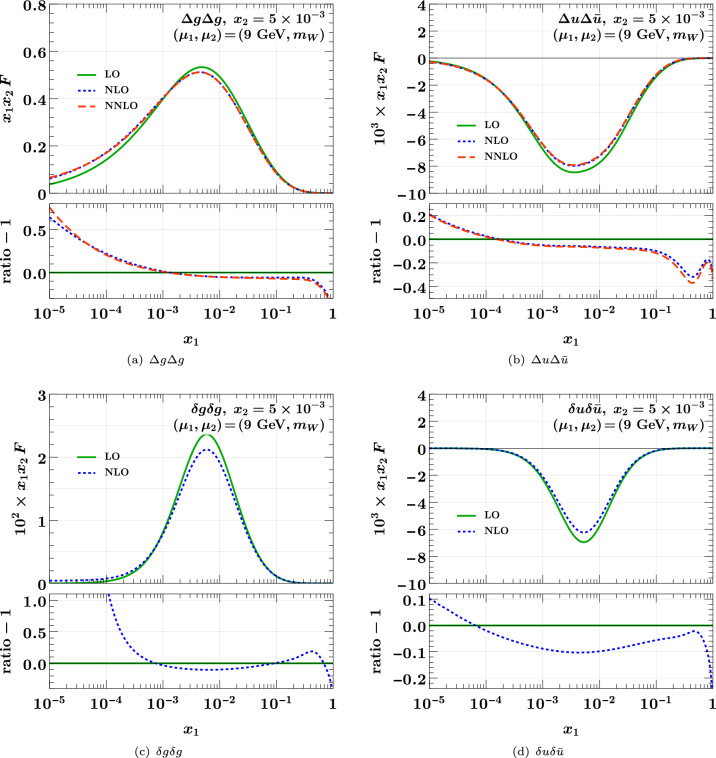
As Fig. 1 but for polarized DPDs. Panels **a** and **b** are for longitudinal, panel **c** for linear, and panel **d** for transverse polarization

We show the effect of the higher-order DGLAP evolution for a selection of unpolarized DPDs in Fig. 1. The distributions are given in the top panel of each plot, and their ratios with respect to the leading-order distribution in the lower panel. At small momentum fractions, higher-order evolution has an effect of order $10\%$ on $F_{g g}$, whereas for the other flavor combinations shown in the figure, the effect is of order 1. In all cases, we find that the change from LO to NLO is much larger than the rather moderate change from NLO to NNLO.

A selection of polarized DPDs is shown in Fig. 2. For linear gluon and transverse quark polarization, we have only implemented evolution at LO and NLO. We find that the NLO corrections to evolution introduce effects of order $10\%$ for $\Delta u\Delta {\bar{u}}$ and $\delta u \delta {\bar{u}}$ in different regions of $x_1$, where as for $\Delta g \Delta g$ they reach $70\%$ at small $x_1$. For $\delta g \delta g$, one obtains a huge relative difference between NLO and LO evolution in the limit $x_1 \ll x_2$. This is because for $z\rightarrow 0$ the splitting function $P_{\delta g \delta g}(z)$ vanishes like *z* at LO but grows like 1/*z* at NLO [69]. At both orders, the evolved distribution at $x_1 \ll x_2$ remains however small compared with its peak around $x_1 \sim x_2$.

**Fig. 3:**
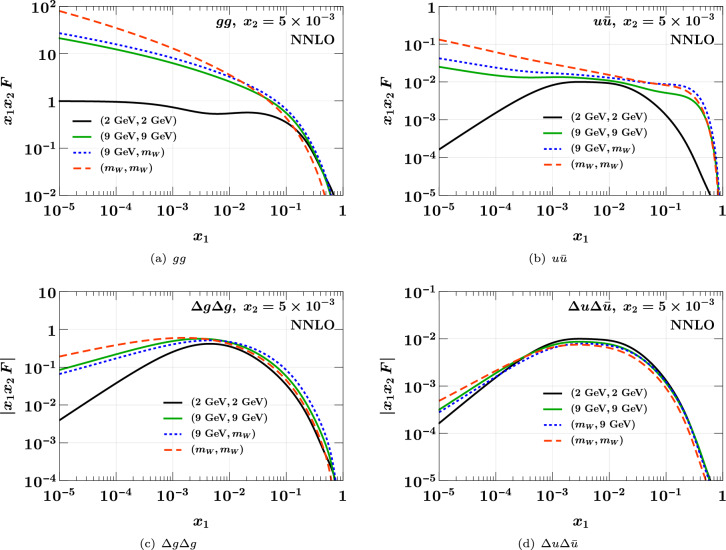
Comparison of DPDs at different scales. The starting conditions at $(\mu _1, \mu _2) = (2 \,{\textrm{GeV}}, 2 \,{\textrm{GeV}})$ and $(n_{1}, n_{2}) = (3, 3)$ are the same as those used for the previous two figures. At higher scales, the number of active flavors is $n_{i} = 3$ for $\mu _i = 9 \,{\textrm{GeV}}$ and $n_{i} = 5$ for $\mu _i = m_W$. Evolution and flavor matching are carried out at the highest available order

As is the case for PDFs, DPDs are not observables, and distributions evolved at different orders are combined with parton-level cross sections computed at different orders. Nevertheless, the strong evolution effects we observed in several channels and kinematic regions suggest that NLO corrections in double parton scattering can be of substantial size.

In Fig. 3, we illustrate the scale dependence of DPDs, using evolution at NNLO and flavor matching at the highest available order (NNLO for unpolarized partons and NLO for polarized ones). We show the distributions at the starting point $(\mu _1, \mu _2) = (2 \,{\textrm{GeV}}, 2 \,{\textrm{GeV}})$, and after evolution to different combinations of the scales $\mu _i = 9 \,{\textrm{GeV}}$ or $\mu _i = m_W$ for the two partons, with associated flavor numbers $n_{i} = 3$ in the first and $n_{i} = 5$ in the second case. For unpolarized partons, we find that the effect of evolution from $\mu _1 = \mu _2 = 2 \,{\textrm{GeV}}$ to $9 \,{\textrm{GeV}}$ is huge at small *x*, turning a shape that is flat or increasing with *x* at the low scale into a shape decreasing with *x* at the higher scale. Subsequent evolution to $m_W$ for one or both partons has a significant, although less dramatic, effect. For polarized partons, evolution is generally weaker, and we find DPDs increasing with *x* at small *x* even when both partons are taken at the high scale $m_W$.

## Interpolation and evolution of DPDs in ChiliPDF

In this section we sketch the mathematical framework underlying ChiliPDF, summarizing the relevant methods presented in Ref. [55] and extending them from PDFs to DPDs. In particular, we give explicit formulae for interpolation and integration, and for the discretization of the DGLAP evolution equations. A detailed account of Chebyshev interpolation and its applications can be found in Ref. [77].^3^

### Chebyshev interpolation

We start by reviewing Chebyshev interpolation of a function *f*(*t*) on the interval $t \in [-1, 1]$. The function is discretized on a grid consisting of the so-called *Chebyshev points*, which for given *N* read

10 $$\begin{aligned} t_i = \cos \Bigl ( \frac{i \pi }{N} \hspace{0.83328pt}\Bigr ) \quad \text {with} \ i = 0, \ldots , N. \end{aligned}$$

These are the points where the *N*th Chebyshev polynomial of the first kind, $T_N(t)$, assumes its maxima $+1$ and minima $-1$. They form a descending series from $t_0 = 1$ to $t_N = -1$ and satisfy the symmetry property $t_{N-i} = - t_i$. We call the set of Chebyshev points a *Chebyshev grid*.

A sufficiently smooth function *f*(*t*) on $t \in [-1, 1]$ can be approximated by the *Chebyshev interpolant* $p_N(t)$. This is a series of Chebyshev polynomials $T_j(t)$ with $j = 0, \ldots , N$ whose expansion coefficients are such that one has $p_N(t_i) = f(t_i)$ at the Chebyshev points. For details see Eqs. (2.6) to (2.8) in Ref. [55].

***Barycentric formula.*** A simple and efficient way to compute the Chebyshev interpolant without having to evaluate Chebyshev polynomials is given by the *barycentric formula*

11 $$\begin{aligned} p_N(t) = \sum _{i=0}^{N} f(t_i) \, b_i(t) \end{aligned}$$

with the barycentric basis functions

12 $$\begin{aligned} b_i(t) = \beta _i \, \frac{(-1)^{i}}{t - t_i} \Bigg / \sum _{j=0}^{N} \beta _j \, \frac{(-1)^{j}}{t - t_j} , \end{aligned}$$

where $\beta _0 = \beta _N = 1/2$ and $\beta _i = 1$ otherwise. Even though it is not directly evident from Eq. (12), the $b_i(t)$ are polynomials of order *N*. The number of operations for evaluating the barycentric formula scales linearly with *N*. The barycentric formula is found to be numerically stable in the interpolation interval (however, this is *not* the case for extrapolating the function *f*(*t*) outside this interval, see chapter 5 of Ref. [77]).

***Accuracy estimate.*** A computationally inexpensive estimate for the accuracy of Chebyshev interpolation is obtained by interpolating *f*(*t*) on the Chebyshev grid *without* the end points $t_0$ and $t_{N}$. The resulting interpolant $q_{N-2}(t)$ is a polynomial of order $N - 2$ and can be computed with an adapted form of the barycentric formula, see Eqs. (2.23) to (2.27) in Ref. [55]. Comparing $p_{N}(t)$ with $q_{N-2}(t)$ yields an estimate for the interpolation accuracy, i.e. for the difference between $p_{N}(t)$ and *f*(*t*). This estimate was found to work well for PDF interpolation [55], and we will investigate in later chapters how reliable it is in the case of DPDs.

***Integration.*** Using the expansion of *f* in terms of Chebyshev polynomials $T_i(t)$ and the analytic form for integrals of these polynomials, one readily obtains the integration rule

13 $$\begin{aligned} \int _{-1}^1 \textrm{d}t \, f(t) \approx \sum _{i=0}^{N}\, w_i\hspace{0.83328pt}f(t_i) \end{aligned}$$

with weights

14 $$\begin{aligned} w_i = \frac{4 \beta _i}{N}\, \sum _{\genfrac{}{}{0.0pt}{}{j=0}{\text {even}}}^{N}\, \beta _j\, \frac{\cos (j \hspace{0.83328pt}\theta _i)}{1-j^2}. \end{aligned}$$

This integration rule is known as *Clenshaw–Curtis quadrature*. For a detailed discussion of its accuracy (and comparison with Gauss quadrature), we refer to chapter 19 of Ref. [77] and to section 2 of Ref. [55].

An estimate for the integration accuracy can be obtained by using a quadrature rule on the Chebyshev grid without its end points, in full analogy to the procedure described above for interpolation. The result is known as *Fejér’s second rule*. Details are given in Eqs. (2.31) to (2.33) of Ref. [55].

### Interpolation strategy

In this subsection, we consider the scales $\mu _1$ and $\mu _2$ to be fixed and omit them as arguments for brevity. We discuss the discretization of DPDs in the three variables $x_1$, $x_2$, and *y*, which we generically denote by *z*. Depending on the typical dependence of a DPD on *z*, different variable transformations $z \rightarrow u$ are used to map an interval $[z_{\min }, z_{\max }]$ onto a finite interval $[u_{\min }, u_{\max }]$. For the momentum fractions $x_1$ and $x_2$ we always use the transformation

15 $$\begin{aligned} u(x_i) = \ln x_i\quad \text {for } i=1,2, \end{aligned}$$

which corresponds to the choice for interpolating PDFs in Ref. [55]. For both $x_1$ and $x_2$, the interpolation interval has a fixed upper limit $x_{\max } = 1$, whereas the lower limits may be equal or different (but must be nonzero). For the *y* dependence (where $y_{\max }$ may be infinite or finite) we use a number of different transformations, which are presented in Sect. 6.1.

For reasons given below, the *z* intervals are usually split into a few subintervals. On each subinterval, we perform a linear transformation from *u* to a variable $t \in [-1,1]$, which is used for Chebyshev interpolation as described in Sect. 3.1.

Consider a particular subgrid with $N + 1$ points $u_0, \ldots , u_N$, which is mapped by a linear transform onto the Chebyshev grid $t_0, \ldots , t_N$ given in Eq. (10). The corresponding grid points in the physical variable are then given by the inverse variable transformation $z_i = z(u_i)$. Similarly to the PDF case, it is advantageous to interpolate DPDs scaled by their momentum fractions,

16 $$\begin{aligned} {\widetilde{F}}(x_1, x_2, y) = x_1 \hspace{0.83328pt}x_2 \hspace{0.83328pt}F(x_1, x_2, y) , \end{aligned}$$

because this leads to less steep functions in $x_1$ and $x_2$. The interpolation in one variable is given by the barycentric formula

17 $$\begin{aligned} {\widetilde{F}}(z, \ldots ) \approx \sum _{i = 0}^N {\widetilde{F}}_i(\ldots ) \; b_i(u(z)) \quad \text {for}\ z_0 \le z \le z_N , \end{aligned}$$

where the ellipsis denotes the other variables and

18 $$\begin{aligned} {\widetilde{F}}_i(\ldots )&= {\widetilde{F}}(z_i, \ldots ) , \nonumber \\ b_i(u)&= \beta _i \, \frac{(-1)^{i}}{u - u_i} \Bigg / \sum _{j=0}^{N} \beta _j \, \frac{(-1)^{j}}{u - u_j}. \end{aligned}$$

The interpolation of the full DPD is obtained by discretization in both momentum fractions and the interparton distance and reads

19 $$\begin{aligned} {\widetilde{F}}(x_1, x_2, y)&\approx \sum _{i=0}^{N_{x\hspace{-0.83328pt}, 1}} \sum _{j=0}^{N_{x\hspace{-0.83328pt}, 2}} \sum _{k=0}^{N_y} {\widetilde{F}}_{i j k} \nonumber \\&\quad \times b_i\bigl ( \ln (x_1) \bigr ) \, b_j\bigl ( \ln (x_2) \bigr ) \, b_k\bigl ( u(y) \bigr ) , \end{aligned}$$

where $N_{x\hspace{-0.83328pt}, 1}$, $N_{x\hspace{-0.83328pt}, 2}$, and $N_y$ specify the polynomial order of interpolation in each variable, and

20 $$\begin{aligned} {\widetilde{F}}_{i j k}= {\widetilde{F}}(x_{1, i},\hspace{0.83328pt}x_{2, j},\hspace{0.83328pt}y_k) \end{aligned}$$

denotes the values of ${\widetilde{F}}$ on the interpolation grid. Equation (18) reflects that the form of the barycentric basis functions (12) remains unchanged under a linear transform of the interpolation variable. Analogous formulae can be used to interpolate functions derived from DPDs, such as the Mellin convolutions of DPDs with an integral kernel (see Sect. 3.3).

Note that we interpolate ${\widetilde{F}}(x_1, x_2, y)$ on a full rectangle in the $(x_1, x_2)$ plane, given by $x_1 \in [x_{1, \min }, 1]$ and $x_2 \in [x_{2, \min }, 1]$. This includes the region $x_1 + x_2 > 1$ where the DPD is zero. In this way, we can use the same interpolation grid in $x_1$ for all values of $x_2$ and vice versa, which in turn permits a simple implementation of DGLAP evolution for the two partons. The implication of this choice on the interpolation accuracy is discussed in Sect. 4.

Chebyshev interpolation is “global” in the sense that the interpolant at a given point *z* depends on the function values at *all* points $z_i$ in the interpolation interval. For grids with many points, this can lead to an accumulation of rounding errors, especially in regions of *z* where the function is much smaller than elsewhere in the interval. To limit this effect, it is advantageous to split the full *z* domain into several subintervals and to use the barycentric formula (19) separately on each subinterval. In Ref. [55] this was found to substantially improve the interpolation accuracy for PDFs, provided that the number of points in each subinterval does not become too small. We find that the same is true for the interpolation of DPDs in $x_1$ and $x_2$, as well as in *y*.

To specify such a composite grid with *k* individual subgrids, we use the notation

21 $$\begin{aligned}{}[z_0,\hspace{0.83328pt}z_1,\hspace{0.83328pt}\ldots ,\hspace{0.83328pt}z_{k}]_{(p_1,\hspace{0.83328pt}p_2,\hspace{0.83328pt}\ldots ,\hspace{0.83328pt}p_k)} , \end{aligned}$$

where $z_i$ are the subinterval boundaries and $p_i = N_i + 1$ is the number of Chebyshev points in subgrid number *i*. We refer to this as a $(p_1, p_2, \ldots , p_k)$-point grid. Note that neighboring subgrids share their end points, such that the total number of grid points is $p_{\text {total}} = \sum _{i = 1}^k p_i - (k - 1)$.

Splitting the interpolation interval in *y* has the additional benefit that one can use different variable transformations *u*(*y*) for regions with different characteristic *y* dependence, such as the power law behavior from perturbative splitting and an exponential decrease at large distances. Details will be discussed in Sect. 6.1.

### Mellin convolution

Consider now the convolution of a DPD with an integral kernel, which may be a DGLAP evolution kernel, a flavor matching kernel, or a hard-scattering coefficient. The general expression to be computed reads

22 $$\begin{aligned} \bigl ( K \underset{}{\otimes } {\widetilde{F}} \,\bigr ) (x) = \int _x^1 \frac{\textrm{d}z}{z} \, K(z) \, {\widetilde{F}} \left( \frac{x}{z} \right) , \end{aligned}$$

where *x* stands for $x_1$ or $x_2$ and the dependence of the rescaled DPD on all other variables is omitted for now. The kernel *K*(*z*) is appropriately rescaled, given that $g_1 = g_2 \otimes g_3$ implies $h_1 = h_2 \otimes h_3$ with $h_k(z) = z \hspace{0.83328pt}g_k(z)$.

To discretize Eq. (22), we consider for simplicity a single Chebyshev grid with $p_x$ points for $x \in [x_{\min }, 1]$. The result of the convolution $(K \otimes {\widetilde{F}})(x)$ is a function defined in the same *x* interval as ${\widetilde{F}}(x)$ and can hence be interpolated on the same grid. It is thus sufficient to evaluate Eq. (22) at the grid points $x_{i}$,

23 $$\begin{aligned}&\bigl ( K \underset{}{\otimes } {\widetilde{F}} \,\bigr ) (x_{i}) = \int _{x_{i}}^1 \! \frac{\textrm{d}{z}}{z} \, K(z) \, {\widetilde{F}} \left( \frac{x_{i}}{z} \right) \nonumber \\&\quad \approx \int _{x_{i}}^1 \frac{\textrm{d}{z}}{z} \, K(z) \, \sum _{i' = 0}^{p_x - 1} {\widetilde{F}}(x_{i'}) \; b_{i'}\!\left( \ln \frac{x_{i}}{z} \right) , \end{aligned}$$

where in the second step we have approximated ${\widetilde{F}} (x_i/z)$ by its interpolated form. Here and in the following, we use the number of grid points $p_x$ rather than the polynomial order $N_x = p_x - 1$ to specify summation ranges. At the grid points, the convolution is thus obtained by a matrix multiplication

24 $$\begin{aligned} \bigl ( K \underset{}{\otimes } {\widetilde{F}} \,\bigr ) (x_i) \approx \sum _{i' = 0}^{p_x - 1} K_{i i'} \, {\widetilde{F}}(x_{i'}) , \end{aligned}$$

where the *kernel matrix*

25 $$\begin{aligned} K_{i i'} = \int _{x_{i}}^1 \frac{\textrm{d}{z}}{z} \, K(z) \; b_{i'}\!\left( \ln \frac{x_{i}}{z} \right) \end{aligned}$$

can be computed using standard numerical integration techniques (we use an adaptive Gauss–Kronrod routine). If *K*(*z*) contains plus- or $\delta$ distributions, this requires rewriting Eq. (25) as specified in Eqs. (4.5) to (4.10) of Ref. [55].

It is straightforward to generalize the preceding discussion to the case where the interval $[x_{\min }, 1]$ is split into several subintervals with corresponding subgrids. The relation (24) remains valid, with an appropriately generalized definition of the kernel matrix and with $p_x$ being the *total* number of grid points in *x*.

The kernel matrix method can be readily used for flavor matching of DPDs, discretizing the Mellin convolutions with the matching kernels in Eq. (4). It is also a crucial part in DPD evolution, which is presented next.

### DGLAP evolution

We now discuss the evolution of DPDs, starting with evolution in $\mu _1$ at a fixed value of $\mu _2$. The evolution equation for the rescaled DPD reads

26 $$\begin{aligned}&\frac{\textrm{d}}{\textrm{d}\ln \mu _1^2} \, {\widetilde{F}}(x_1,x_2,y; \mu _1,\mu _2)\nonumber \\&\quad = \Bigl [ {\widetilde{P}}(\mu _1) \underset{1}{\otimes } {\widetilde{F}}(x_2,y; \mu _1,\mu _2) \Bigr ](x_1) , \end{aligned}$$

where ${\widetilde{P}}(z; \mu _1) = z \hspace{0.83328pt}P(z; \mu _1)$ is the rescaled splitting function and we omit parton labels and the associated sums for the time being. After discretization on one or several subgrids with a total number of $p_{x\hspace{-0.83328pt},1}$, $p_{x\hspace{-0.83328pt},2}$, $p_y$ points in $x_1$, $x_2$, *y*, the integro-differential equation (26) turns into a linear system of ordinary differential equations in $\mu _1$,

27 $$\begin{aligned} \frac{\textrm{d}}{\textrm{d}\ln \mu _1^2} \, {\widetilde{F}}_{i j k}(\mu _1, \mu _2) = \sum _{i'=0}^{p_{x\hspace{-0.83328pt}, 1} - 1} \, \!\! {\widetilde{P}}_{i i'}(\mu _1) \, {\widetilde{F}}_{i'\hspace{-0.83328pt}j k}^{}(\mu _1, \mu _2) , \end{aligned}$$

where ${\widetilde{F}}_{i j k}(\mu _1, \mu _2)$ is given in Eq. (20) and ${\widetilde{P}}_{i i'}$ is the matrix for the kernel ${\widetilde{P}}(z)$ as defined in Eq. (25) or its generalization to several subgrids. At NNLO accuracy, we have

28 $$\begin{aligned} {\widetilde{P}}_{i i'}(\mu ) =\sum _{k=0}^{2} \biggl [ \frac{\alpha _s(\mu )}{4\pi } \biggr ]^{k+1} \; {\widetilde{P}}_{i i'}^{(k)} , \end{aligned}$$

and after a change of variables from the scales $\mu _i$ to the evolution times

29 $$\begin{aligned} t_i = - \ln \bigl ( \alpha _s(\mu _i) \bigr )\quad \text {for } i=1,2, \end{aligned}$$

we obtain

30 $$\begin{aligned} \frac{\textrm{d}}{\textrm{d}t_1} \, {\widetilde{F}}_{i j k}(t_1, t_2) = \sum _{i'=0}^{p_{x\hspace{-0.83328pt}, 1} - 1} \, K_{i i'}(t_1) \, {\widetilde{F}}_{i'\hspace{-0.83328pt}j k}(t_1, t_2) \end{aligned}$$

with a new kernel matrix given by

31 $$\begin{aligned} K_{i i'}(t) =&\biggl [ \hspace{0.83328pt}\beta _0 + \frac{e^{-t}}{4\pi } \, \beta _1 + \frac{e^{-2 t}}{(4\pi )^2} \, \beta _2 \, \biggr ]^{-1} \; \nonumber \\&\times \biggl [ \hspace{0.83328pt}{\widetilde{P}}_{i i'}^{(0)} + \frac{e^{-t}}{4\pi } \, {\widetilde{P}}_{i i'}^{(1)} + \frac{e^{-2 t}}{(4\pi )^2} \, {\widetilde{P}}_{i i'}^{(2)} \, \biggr ] , \end{aligned}$$

where $\beta _k$ are the expansion coefficients of the QCD $\beta$ function (see Eq. (5.4) in [55] for our normalization conventions).

To solve Eq. (30) we use a Runge–Kutta algorithm with a fixed maximal step size in $t_1$. Notice that the $t_1$ dependence of $K_{i i'}(t_1)$ starts only at NLO, which leads to a more uniform accuracy of the algorithm compared with the direct solution of Eq. (27), where ${\widetilde{P}}_{i i'}(\mu _1)$ changes with $\ln \mu _1$ already at LO. As documented in appendix A of Ref. [55], we find that the use of a higher-order Runge–Kutta algorithm – in particular the 8th order algorithm of Dormand and Prince [78] – greatly improves the accuracy of evolution for a given number $N_{\text {RK}}$ of intermediate time values at which the r.h.s. of the differential equation is evaluated for evolution from one value of $t_1$ to another.

The Runge–Kutta algorithm is also used to compute the transformation (29), solving the differential equation for the running of $\alpha _s^{-1}$ as a function of $\ln \mu$.

Flavor labels and sums have to be added in Eq. (30) as in the original version (1). The mixing between parton flavors under evolution is the same as for PDFs, and correspondingly we solve the evolution equations in the same basis that is used for PDFs in Ref. [55]. For each of the two partons, we thus form the linear combinations

32 $$\begin{aligned}&\Sigma ^{\pm } = \sum _i q_i^\pm , \quad u^\pm - d^\pm , \quad d^\pm - s^\pm ,\nonumber \\&s^\pm - c^\pm , \quad c^\pm - b^\pm , \quad b^\pm - t^\pm , \end{aligned}$$

where $q^\pm = q \pm {\bar{q}}$. The combination $\Sigma ^-$ and all flavor differences then evolve by themselves, and mixing is reduced to the combination $\Sigma ^+$ and the gluon.

The system (30) needs to be solved separately for each combination of the indices *j*, *k* that correspond to the “inactive” variables $x_2$ and *y* during evolution in $t_1$, and for each of the $2 n_{2} + 1$ active flavors of the second parton. This gives a large number of $p_{x\hspace{-0.83328pt}, 2} \; p_y \, (2 n_{2} + 1)$ combinations – typically several thousand. It is much more efficient to apply the Runge–Kutta algorithm not to each of these combinations, but to a set of $p_{x\hspace{-0.83328pt}, 1}$ basis vectors $e_{i}^{j}$ that generates all possible initial conditions on the grid for $x_1$. With the choice of basis $e_{i}^{\hspace{0.83328pt}j} = \delta _{i j}^{}$, evolution from $t_{0 1}$ to $t_1$ requires solving the system

33 $$\begin{aligned} \frac{\textrm{d}}{\textrm{d}t_{1}} \, U_{i j}(t_{1}, t_{0 1}) = \sum _{i' = 0}^{p_{x\hspace{-0.83328pt}, 1} - 1} K_{i i'}(t_{1}) \, U_{i' j}(t_{1}, t_{0 1}) , \end{aligned}$$

with the boundary condition $U_{i j}(t_{0 1}, t_{0 1}) = \delta _{i j}$. The solution of Eq. (30) is then obtained by a matrix multiplication

34 $$\begin{aligned} {\widetilde{F}}_{i j k}(t_{1}, t_{2}) = \sum _{i' = 0}^{p_{x\hspace{-0.83328pt}, 1} - 1} U_{i i'}(t_{1}, t_{0 1}) \, {\widetilde{F}}_{i' j k}^{}(t_{0 1}, t_2) , \end{aligned}$$

where the evolution matrix $U_{i i'}(t_{1}, t_{0 1})$ acts as the discretized version of the Green function for the original evolution equation (26). With flavor labels reinstated, Eq. (33) is solved separately for the evolution kernels of the different channels (i.e. for $q_i^{+} - q_j^{+}$, $q_i^{-} - q_j^{-}$, $\Sigma ^{-}$, and the coupled system of $\Sigma ^{+}$ and the gluon).

This approach is also attractive in the PDF case when a large set of different PDFs is evolved simultaneously, and it is for instance used in the PDF evolution codes APFEL [49] and EKO [53], with $U_{i i'}(t_{1}, t_{0 1})$ being called an evolution operator and an evolution kernel operator, respectively. In ChiliPDF, the evolution of a DPD in one scale is implemented in the same way as the evolution of a set of PDFs.

The number of operations for evaluating Eq. (34) for all indices *i*, *j*, *k* and flavors for the second parton scales like $(p_{x\hspace{-0.83328pt}, 1})^2 \; p_{x\hspace{-0.83328pt}, 2} \; p_y \, (2 n_{2} + 1)$, and the number of operations for solving the evolution equation (33) scales like $(p_{x\hspace{-0.83328pt}, 1})^3 \, N_{\text {RK}}$ with $N_{\text {RK}}$ introduced below Eq. (31). The number of grid points for the interpolation in the momentum fractions is therefore a major factor determining the computational speed of DPD evolution.

The preceding discussion is trivially extended to evolution in the scale $\mu _2$ at fixed $\mu _1$. To evolve from $(\mu _{0 1}, \mu _{0 2})$ to $(\mu _{1}, \mu _{2})$, we first evolve from $(\mu _{0 1}, \mu _{0 2})$ to $(\mu _{1}, \mu _{0 2})$ and from there to $(\mu _{1}, \mu _{2})$. We verified that evolving to $(\mu _{0 1}, \mu _{2})$ in the first step leads to the same final result with very high numerical accuracy (see also Sect. 5.1).

Let us briefly sketch how the initial conditions for DPD evolution are set up in ChiliPDF. In a first step, the values of $F_{a_1 a_2}\bigl (x_1, x_2, y; \mu (y), \mu (y)\bigr )$ for a selected number $n_{0}$ of active flavors are computed on the grid points in $x_1$, $x_2$ and *y*, either from pre-defined functions for the splitting form (7), or from functions implementing a product ansatz like the one in (8), or from functions entirely provided by the user. As is adequate for the physics, the input scale $\mu (y)$ may depend on *y* as specified by the user. For each grid point $y_k$, the discretized DPD is then evolved to a pair of scales $(\mu _{0}, \mu _{0})$ that is independent of *y* and stored as an initial condition. After this, all evolution calls can use the same set of evolution matrices for all grid points $y_k$, which significantly reduces computing time. One may also add two DPDs (after evolution to a common scale pair), which is for instance needed for calculating $F^{\text {int}} + F^{\text {spl}}$.

In the procedure just described, the flavor number $n_{0}$ is equal for both partons. DPDs with larger values of $n_{1}$ or $n_{2}$ are then obtained by flavor matching, with initial conditions at the pair of scales $(\mu _1, \mu _2)$ where the matching is carried out. More detail is given in Sect. 5.1.

## Interpolation of DPDs in both momentum fractions

As shown in the previous section, our approach to interpolating DPDs in $x_1$ and $x_2$ is a straightforward two-dimensional generalization of the method presented in Ref. [55] for the interpolation of PDFs in *x*. In the present section, we study the performance of this approach. Let us point out some particularities of the DPD case:

1. To limit computation time, interpolation grids should not have too many points, while still giving numerically reliable results. For PDFs one can typically afford denser grids, and the demands on interpolation accuracy are often higher than for DPDs.
2. The DPD is a non-analytic function of $x_1$ at the point $x_1 = 1 - x_2$ because it vanishes identically above that value. Since we interpolate on the same grid in $x_1$ for all values of $x_2$, this singular point is generally in the middle of an interpolation interval. Polynomial interpolation is not very accurate in the vicinity of such a point, but we will see that this effect is in general relatively mild.
3. The perturbative splitting mechanism gives a dependence on $x_1$ that is qualitatively different from the *x* dependence of a PDF. In some channels, the splitting contribution (7) goes to zero for $x_1 \ll x_2$ and thus results in a *strong* rise of the DPD as a function of $x_1$.

Of course, the last two points also apply to interpolation in $x_2$ at a given $x_1$.

In the following, we will study the interpolation accuracy for DPDs that have a known analytic expression. To this end, we simplify the forms (8) and (7) to

35 $$\begin{aligned}&{\widehat{F}}_{a_1 a_2}^{\hspace{0.83328pt}\text {int}\hspace{0.83328pt}(r)}(x_1,x_2)\nonumber \\&\quad = \frac{(1 - x_1 - x_2)^r}{(1 - x_1)^r (1 - x_2)^r} \; f_{a_1}(x_1)\, f_{a_2}(x_2) \end{aligned}$$

and

36 $$\begin{aligned}&{\widehat{F}}_{a_1 a_2}^{\hspace{0.83328pt}\text {spl}}(x_1,x_2)\nonumber \\&\quad = V^{(1)}_{a_1 a_2, a_0} \biggl ( \frac{x_1}{x_1 + x_2} \biggr ) \, \frac{f_{a_0}(x_1 + x_2)}{x_1 + x_2} , \end{aligned}$$

respectively. For the PDFs, we take the simple parameterizations of the Les Houches benchmark PDFs given in section 1.32 of Ref. [79] and section 4.4 of Ref. [80].

Corresponding to the typical use case, we take the same interpolation grids for $x_1$ and $x_2$ (a situation in which different grids are useful is encountered in Sect. 7). We consider three composite grids of the form

37 $$\begin{aligned}&[10^{-5}, 5 \times 10^{-3}, 0.5, 1]_{(p,\, p,\, p)}\nonumber \\&\quad \text {with } p = {\left\{ \begin{array}{ll} 12 &{} (\text {coarse }x \text { grid)} \\ 16 &{} (\text {medium }x \text { grid)} \\ 24 &{} (\text {fine }x \text { grid)} \end{array}\right. } \end{aligned}$$

where the shorthand notation from Eq. (21) has been used. We find that for the given *x* range, three subgrids with the above interval limits generally give the best accuracy for a given total number of points. As a measure for the quality of interpolation, we use the relative accuracy defined as

38 $$\begin{aligned}&\text {relative accuracy}\nonumber \\&\quad = \bigl | \text {interpolated result} / \text {exact result} - 1 \bigr |. \end{aligned}$$

In the following, this interpolation accuracy will be shown as a function of $x_1$ for fixed values of $x_2$. The latter are *not* grid points, so that interpolation is performed for both momentum fractions.

### Interpolation of the product form

We start with the product form (35) for the three values $r = 0, 1, 2$ of the power in the phase space factor. The case $r = 0$ corresponds to a “naive” product form with a discontinuity at $x_1 + x_2 = 1$ that is not very plausible on physics grounds. In fact, evolution to higher scales quickly changes such a discontinuity to a steep but continuous decrease. We nevertheless include the case $r = 0$ here, in order to see how our method performs in this limiting case. The following plots show the $u {\bar{u}}$ distribution; we verified that for other parton combinations the interpolation accuracy is not significantly better or worse.

**Fig. 4:**
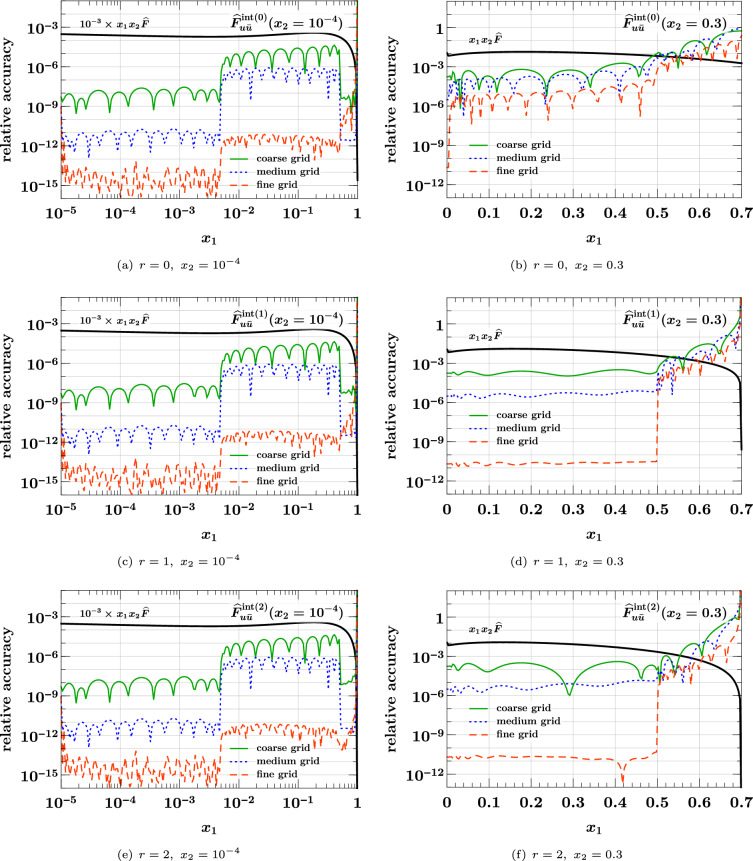
Relative accuracy (38) for interpolation in $x_1$ and $x_2$, evaluated for the product form ${\widehat{F}}_{u {\bar{u}}}^{\hspace{0.83328pt}\text {int}\hspace{0.83328pt}(r)}(x_1,x_2)$ of Eq. (35) with $r = 0,1,2$. The interpolation grids are specified in Eq. (37). Here and in similar plots, the exact result that is being interpolated is shown in black

In the left panels of Fig. 4 we see that all three grids yield an excellent interpolation accuracy at small momentum fractions. Not surprisingly, there is little dependence on the power *r* in this region, given that the phase space factor is very close to 1 for $x_1, x_2 \ll 1$. In the right panels of the figure, we see that a degradation of the relative accuracy sets in as $x_1$ approaches its kinematic limit $1 - x_2 = 0.7$, and that the degradation is stronger for smaller *r*. Nevertheless, the accuracy is better than 1% as long as the distance $1 - (x_1 + x_2)$ from the kinematic threshold is above 0.15 for $r=1,2$ and above 0.2 for $r=0$.

**Fig. 5:**
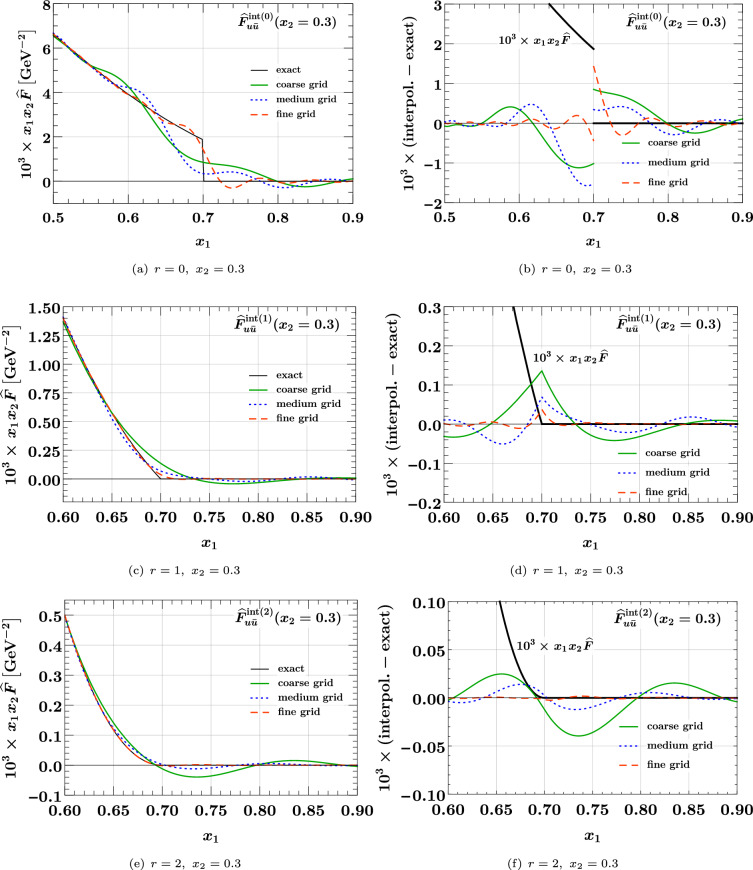
Interpolation of DPDs at large momentum fractions. The DPDs considered are the same as in the right panels of Fig. 4. The plots on the left compare the exact and interpolated forms of the DPDs. The plots on the right show the difference between the interpolated and the exact values, along with the exact form. Notice that the plots in the top row have a different $x_1$ range than the remaining ones

The plots in Fig. 5 show the $x_1$ region around the point where the DPD has a non-analytic behavior, which is at $x_1 = 0.7$ in this example. In these plots, we include the region $x_1 > 1 - x_2$, where the interpolated DPD oscillates around its true value zero. Note that this region is included when one computes a Mellin convolution for the DPD with the kernel matrix method described in Sect. 3.3. However, the effect of oscillations around the true value will partially cancel out in convolution integrals.

We see that for $r=0$, one obtains a rather poor approximation of the DPD in the range $x_1 \in [0.6, 0.8]$ with the coarse or medium grid. In situations where this region is deemed important, one should take a dense grid and check the reliability of the results by comparing with an even denser grid. Of course, this is only needed in the relevant subinterval (i.e. for $x_1 \in [0.5, 1]$ in our example) and hence entails only a moderate increase in the total number of grid points. For $r=1$, where the DPD is continuous but has a discontinuous first derivative at $x_1 = 0.7$, we see that the fine grid performs very well, and for $r=2$ already the medium grid yields a good approximation of the DPD.

### Interpolation of the splitting form

We now turn to the interpolation of the splitting form. We see in Eq. (36) that it decreases almost as fast as a PDF for $x_1 + x_2 \rightarrow 1$, so that according to the results of the previous subsection one does not expect particular problems with interpolation in that region. This is confirmed by our numerical studies.

**Fig. 6:**
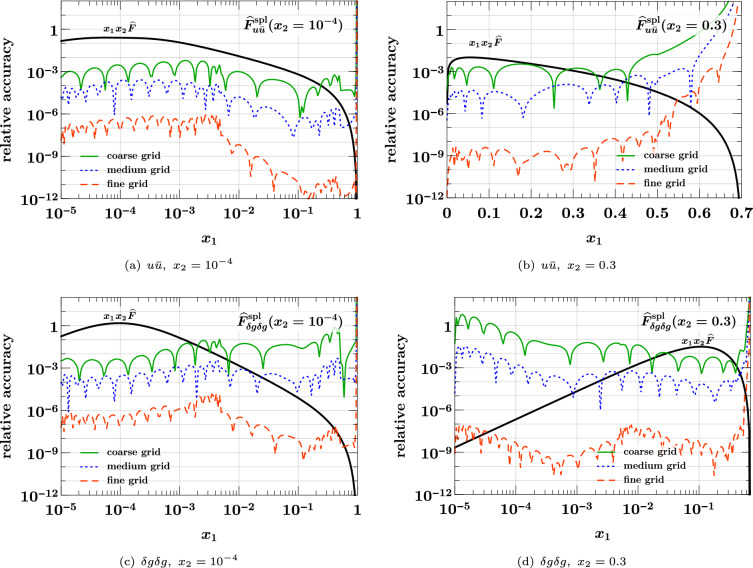
Relative accuracy (38) for interpolation in $x_1$ and $x_2$, evaluated for the splitting form ${\widehat{F}}_{a_1 a_2}^{\hspace{0.83328pt}\text {spl}}(x_1,x_2)$ of Eq. (36). The interpolation grids are specified in Eq. (37). The plots in the top row are for an unpolarized $u {\bar{u}}$ pair, and those in the bottom row for a pair of linearly polarized gluons. Notice that in panel (**d**) a logarithmic scale for $x_1$ is used to plot the DPD at $x_2 = 0.3$.

The shape of the splitting form is strongly influenced by the end-point behavior of the splitting kernel $V^{(1)}_{a_1 a_2, a_0}$, and in the following we show results for two qualitatively different cases:

39 $$\begin{aligned} V^{(1)}_{u {\bar{u}}, g}(z)&= T_F \bigl [ z^2 + (1 - z)^2 \bigr ] , \nonumber \\ V^{(1)}_{\delta g \delta g, g}(z)&= 2 C_A \, z (1 - z). \end{aligned}$$

Whereas the first kernel is finite for $z \rightarrow 0$ and $z \rightarrow 1$, the second one goes to zero in both limits. This implies a strong decrease of ${\widehat{F}}_{\delta g \delta g}^{\hspace{0.83328pt}\text {spl}}(x_1, x_2)$ for both $x_1 \ll x_2$ and $x_1 \gg x_2$. Other splitting kernels diverge at the end points – namely when an unpolarized gluon produced by the splitting becomes soft – and we verified that the interpolation accuracy for these channels is not worse than the one for $u {\bar{u}}$.

We see in the top row of Fig. 6 that already the medium grid yields a very good interpolation as long as the momentum fractions are not too close to the kinematic limit. At small $x_1$ and $x_2$, the accuracy is somewhat less good than for the product form in Fig. 4, which can be understood because the splitting form has more structure in that region. This is also seen for the interpolation of ${\widehat{F}}_{\delta g \delta g}^{\hspace{0.83328pt}\text {spl}}$ at small momentum fractions in panel (c), where the accuracy of the medium grid is at the permille level. In panel (d) we see that the linear decrease of the distribution for $x_1 \ll x_2$ requires the fine grid for a satisfactory interpolation at the smallest $x_1$, where the DPD is many orders of magnitude smaller than its maximum value. On the other hand, the medium grid still performs well for $x_1 > 10^{-4}$. We note that the coarse grid does not give a good accuracy in any of the cases considered here, and it is entirely unreliable in the case of panel (d). We conclude that at least 16 points per subgrid should be taken, unless one considers only unpolarized DPDs and has only very moderate accuracy requirements.

### Estimating the interpolating accuracy

Let us finally study the reliability of the accuracy estimate for interpolation described in Sect. 3.1, given by

40 $$\begin{aligned}&\text {relative accuracy estimate}\nonumber \\&\quad = \biggl | \, \frac{\text {value interpolated without end points}}{\text {value interpolated with end points}} - 1 \, \biggr |. \end{aligned}$$

**Fig. 7:**
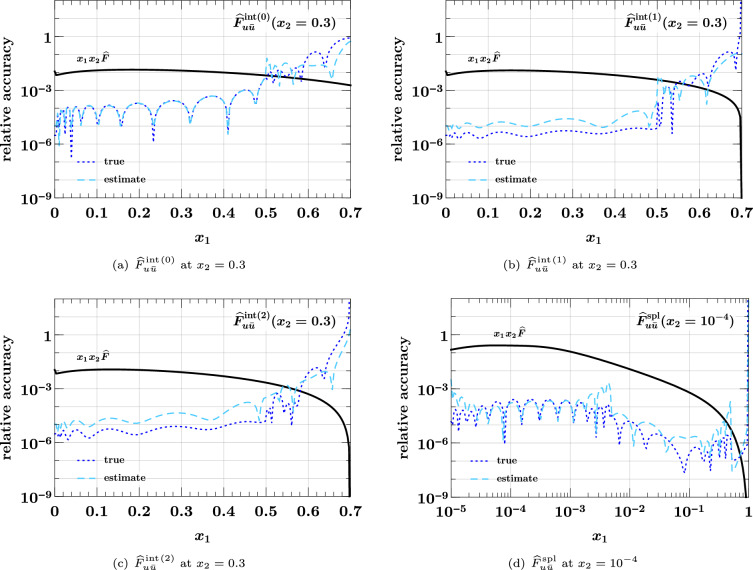
True interpolation accuracy in $x_1$ and $x_2$ compared with its estimate (40). All values refer to the medium *x* grid in Eq. (37). The DPDs used are specified in Eqs. (35) and (36)

In Fig. 7, we compare this estimate with the true relative accuracy (38) for the medium *x* grid in Eq. (37). We see that the estimate is typically not more than two orders of magnitude away from the true accuracy (except around points where the error estimate or the true error has a zero crossing). Somewhat surprisingly, the estimate almost coincides with the true accuracy in some cases, as seen in Fig. 7a. Closer inspection reveals that the estimate tends to be above the true accuracy close to subinterval end points; this is expected because in that region interpolation without the end points is particularly bad by construction. We find a qualitatively similar picture for the accuracy estimate in other kinematic settings, and for the other two grids in Eq. (37). Although not perfectly accurate in all regions, the method based on Eq. (40) provides a good and easy-to-compute indicator for the quality of interpolation.

## Evolution and flavor matching of DPDs

In this section, we demonstrate the accuracy of DGLAP evolution and flavor matching for DPDs with the methods presented in Sects. 3.3 and 3.4. As a default setting for DPD evolution, we take a maximal step size of $h = 0.22$ for the evolution time (29) in the Runge–Kutta method. We use the 8th order algorithm of Dormand and Prince [78], where each Runge–Kutta step is divided into 13 substeps for which the r.h.s. of the evolution equation is evaluated.

As initial conditions we take the splitting form (7) evaluated with the Les Houches benchmark PDFs of Refs. [79, 80]. Following the settings in these references, we take

41 $$\begin{aligned} \mu _0 = \sqrt{2} \,{\textrm{GeV}}\end{aligned}$$

as initial scale with $n_{} = 3$ active flavors for both partons. The evolution accuracy is independent of *y*, and we fix this variable to the value $y = b_0 / \mu _0 \approx 0.794 \,{\textrm{GeV}}^{-1}$. Unless specified otherwise, flavor matching is carried out at the canonical scales $\mu _q = m_q$, with the quark masses $m_c = \sqrt{2} \,{\textrm{GeV}}$, $m_b = 4.5 \,{\textrm{GeV}}$, and $m_t = 175 \,{\textrm{GeV}}$. The strong coupling at the initial scale is taken to be $\alpha _s(\mu _0) = 0.35$. Throughout this section, we consider unpolarized partons and use evolution and flavor matching at NNLO. We specify the scales and the number of active flavors for the two partons in the form $(\mu _1, \mu _2)$ and $(n_{1}, n_{2})$.

Guided by the studies in Sect. 4, we interpolate both $x_1$ and $x_2$ on the grid

42 $$\begin{aligned}{}[10^{-5}, 5 \times 10^{-3}, 0.5, 1]_{(16, 16, 24)} . \end{aligned}$$

This corresponds to the medium grid in Eq. (37), except for the third subinterval, where we afford a finer grid for improved interpolation accuracy at large momentum fractions.

### Path independence of evolution and flavor matching

For a given heavy quark flavor *q*, we apply the matching equations (4) at a fixed scale $\mu _{q}$, so that in the $(\mu _1, \mu _2)$ plane we match at the line $(\mu _{q}, \mu _2)$ for the first parton and at the line $(\mu _1, \mu _{q})$ for the second one. We show in Appendix A that, under these conditions, the result of evolution and flavor matching between two points in the $(\mu _1, \mu _2)$ plane is independent of the evolution path, including the order in which the different flavor matching steps are carried out.

**Fig. 8:**
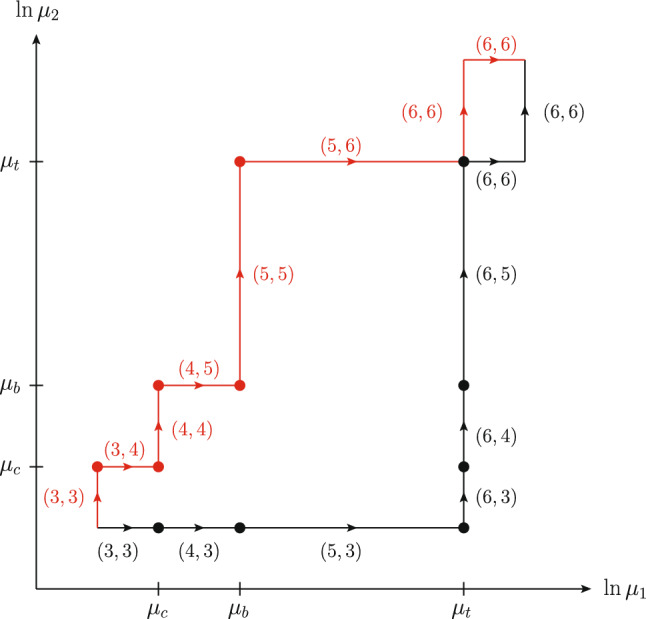
Two paths for evolution and flavor matching in the $(\mu _1, \mu _2)$ plane. Next to each line segment, we specify the numbers $(n_{1}, n_{2})$ of active flavors for which the DPD is evolved. Flavor matching is performed at the points marked by dots. The path in black is the ChiliPDF default for matching from (3, 3) to (6, 6) and for subsequent evolution up to the final scale pair

**Fig. 9:**
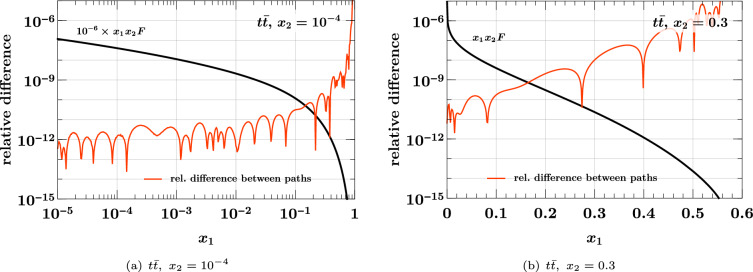
Relative difference between the $t {\bar{t}}$ distribution obtained by evolving and matching along one of the two paths in Fig. 8. Both paths lead from $(1 \,{\textrm{GeV}}, 1 \,{\textrm{GeV}})$ with (3, 3) flavors to $(500 \,{\textrm{GeV}}, 1000 \,{\textrm{GeV}})$ with (6, 6) flavors. The distributions at $(1 \,{\textrm{GeV}}, 1 \,{\textrm{GeV}})$ are obtained by evolving backwards from the starting conditions at $(\mu _0, \mu _0)$ specified at the beginning of the section

We verify this numerically by comparing the results of evolving and matching from $(1 \,{\textrm{GeV}}, 1 \,{\textrm{GeV}})$ with (3, 3) flavors to $(500 \,{\textrm{GeV}}, 1000 \,{\textrm{GeV}})$ with (6, 6) flavors along the two paths shown in Fig. 8. The path in black is the default taken in ChiliPDF when matching from 3 to 6 flavors for both partons and then evolving to the final pair of scales. The staircase-like path in red can be selected by subsequent evolution calls for each line segment. Each point marked by a dot in the figure corresponds to the initial condition of the DPD with given flavor numbers $(n_{1}, n_{2})$ in ChiliPDF. For any scale pair $(\mu _1, \mu _2)$, the DPD with these flavor numbers is computed by evolution from this initial condition.

The results of evolving and matching along the two specified paths are in excellent agreement, as illustrated in Fig. 9 for the $t {\bar{t}}$ distribution, for which the differences are largest among all flavor combinations. The agreement deteriorates close to the kinematic limit $x_1 = 1 - x_2$ but remains small compared with the interpolation accuracy in that region. The choice for a particular evolution path in our implementation is therefore of no consequence for the accuracy.

### Evolution and flavor matching accuracy

We now study evolution and flavor matching to higher scales, using the initial conditions specified at the beginning of this section. To assess the accuracy, we use three settings:

1. our default setting with the grid $$[10^{-5}, 5 \times 10^{-3}, 0.5, $$$$ 1]_{(16, 16, 24)}$$ and a maximal step size $$h = 0.22$$ for the Runge–Kutta algorithm,
2. a finer grid $[10^{-5}, 5 \times 10^{-3}, 0.5, 1]_{(40, 40, 40)}$ with the same maximal step size $h = 0.22$,
3. the same finer grid with a smaller maximal step size $h = 0.0088$.

The difference between settings 1 and 2 is taken as an estimate of the error from discretization in $x_1$ and $x_2$ with our default grid, and the difference between settings 2 and 3 is taken as an estimate of the error due to the Runge–Kutta algorithm.

**Fig. 10:**
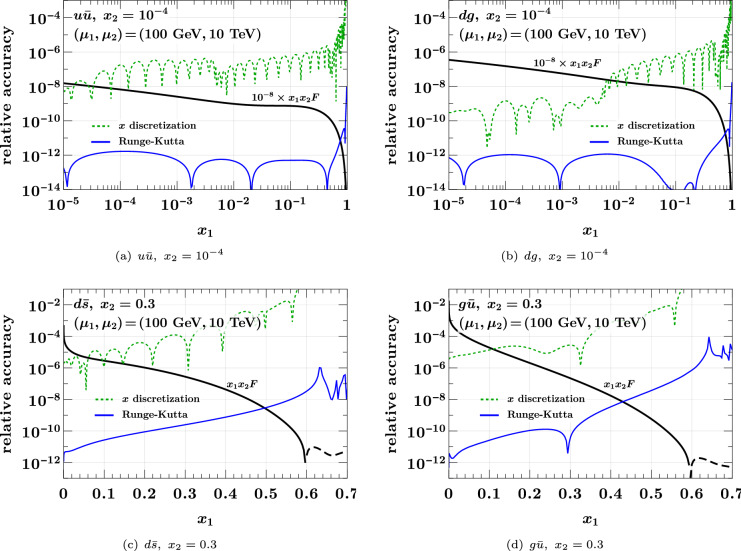
Relative Runge–Kutta and discretization errors for DPD evolution and flavor matching from scales $(\mu _0, \mu _0)$ with (3, 3) flavors to $(100\,{\textrm{GeV}}, 10\,{\textrm{TeV}})$ with (5, 6) flavors. The initial conditions are specified at the beginning of the section

These errors are shown in Fig. 10 for evolution and matching to $(100\,{\textrm{GeV}}, 10\,{\textrm{TeV}})$ with (5, 6) active flavors. The plots are for selected parton combinations; we verified that the errors for the other combinations are of similar size or even smaller. We see that the Runge–Kutta errors are always negligible compared with the errors from discretization. The latter are of similar size as (or even smaller than) the interpolation errors for the initial conditions, which are shown for $u {\bar{u}}$ in Fig. 6. Evolution to high scales does therefore *not* degrade the overall accuracy of DPDs with our method. Only close to the kinematic limit does the relative error from discretization become large. With the chosen grid, it remains below 1% for $1 - (x_1 + x_2)$ above 0.2.

**Fig. 11:**
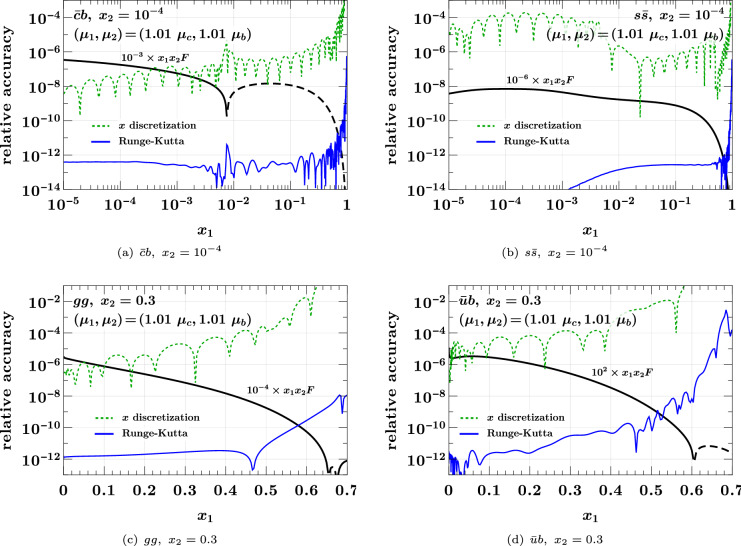
As Fig. 10, but for different flavor combinations evolved and matched to scales $(1.01 \hspace{0.83328pt}\mu _c, 1.01 \hspace{0.83328pt}\mu _b)$ with (4, 5) flavors. A dashed black line indicates that the interpolated function is negative

In Fig. 11, we show the errors for evolution and matching to $(1.01 \hspace{0.83328pt}\mu _c, 1.01 \hspace{0.83328pt}\mu _b)$ with (4, 5) flavors, i.e. to scales just slightly above the matching scales for the final flavor numbers. The accuracy remains excellent, even for DPDs with charm as first or bottom as second parton, which are still tiny at the chosen scales.

### Backward evolution accuracy

A stringent way to test the accuracy of an evolution algorithm is backward evolution from large to small scales. Whilst small inaccuracies of distributions at a given scale are diminished when evolving to higher scales, they are amplified by backward evolution. In the following, we adapt the backward evolution test performed for PDFs in Ref. [55] to the case of DPDs.

We start with the three-flavor input DPD specified at the beginning of this section, match to 4 flavors at the input scales $(\mu _0, \mu _0)$, and then evolve to $(\mu _{\text {low}}, \mu _{\text {low}})$ with $\mu _{\text {low}}= m_b/2 = 2.25 \,{\textrm{GeV}}$. At this point, we match from 4 to 5 flavors for both partons. Since the matching scale differs from $m_b$, all DPDs are nonzero at that point, including the ones with bottom quarks or antiquarks. We take these distributions as starting conditions for evolution with 5 flavors, evolving

1. from $(\mu _{\text {low}}, \mu _{\text {low}})$ to $(\mu _{\text {high}}, \mu _{\text {high}})$ with $\mu _{\text {high}}= 1 \,{\textrm{TeV}}$ in step 1,
2. from $(\mu _{\text {high}}, \mu _{\text {high}})$ back to $(\mu _{\text {low}}, \mu _{\text {low}})$ in step 2.

We use our default value of $h = 0.22$ for the maximal Runge–Kutta step size.

**Fig. 12:**
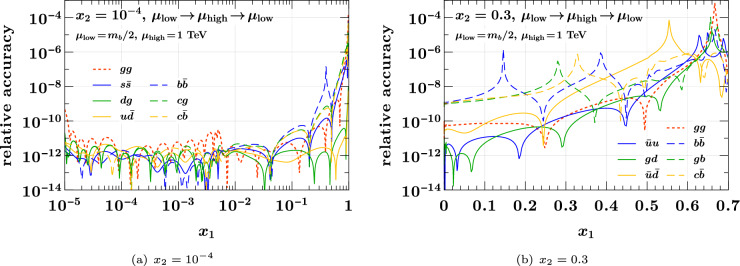
Relative accuracy of evolving DPDs from $\mu _{\text {low}}= m_b /2$ to $\mu _{\text {high}}= 1\,{\textrm{TeV}}$ and back to $\mu _{\text {low}}$ for both partons. Details are given in the text

The relative difference between the input to the first step and the output of the second step is sensitive to the accuracy of forward *and* backward evolution. In Fig. 12, this quantity is shown for selected parton combinations; the accuracy is not worse for other combinations. We find that backward evolution is very precise, with the relative errors in our exercise being below $10^{-6}$ over most of the phase space. This also holds for distributions containing bottom quarks or antiquarks, which have zero crossings at the scale $\mu _{\text {low}}$ where the ratio shown in the figure is evaluated.

We note that backward evolution is inherently more demanding for two scales than for a single scale. Indeed, step 2 of our study corresponds to backward evolution first from $(\mu _{\text {high}}, \mu _{\text {high}})$ to $(\mu _{\text {low}}, \mu _{\text {high}})$ and then from $(\mu _{\text {low}}, \mu _{\text {high}})$ to $(\mu _{\text {low}}, \mu _{\text {low}})$. Inaccuracies accumulated in the first substep are amplified in the second one. We repeated the above exercise for evolution in only one scale, evolving from $(\mu _{\text {low}}, \mu _{\text {low}})$ up to $(\mu _{\text {high}}, \mu _{\text {low}})$ and then down to $(\mu _{\text {low}}, \mu _{\text {low}})$. We find that indeed the accuracy is even higher in that case, by one or two orders of magnitude in some kinematic regions.

**Table 1:** Variable transformations suitable for interpolation in *y*. The parameters $\alpha$ and *m* must be positive. All transformation satisfy $u \le 0$, $y(0) = \infty$, and $\textrm{d}y / \textrm{d}u > 0$ for $u \in [-1,0]$. We abbreviate $L(u) = \ln \bigl ( 1 / |u| \bigr )$

No.	${}-u(y)$	*y*(*u*)	$y(-1)$	$\textrm{d}y / \textrm{d}u$
1	$y^{-\alpha }$	$|u|^{- 1 / \alpha }$	1	$\frac{1}{\alpha } \, |u|^{\hspace{0.83328pt}-(1+\alpha )/\alpha }$
2	$\exp \Bigl [- \frac{1}{4} \hspace{0.83328pt}(m^2 y^2 + m y) \Bigr ]$	$\frac{1}{2 m} \, \bigl (\sqrt{16 L(u) + 1} - 1\bigr )$	0	$\frac{4}{m} \, |u|^{-1} \, \bigl (16 L(u) + 1\bigr )^{-1/2}$
3	$\exp \Bigl [-\frac{1}{4} \hspace{0.83328pt}m y \Bigr ]$	$\frac{4}{m} \hspace{0.83328pt}L(u)$	0	$\frac{4}{m} \hspace{0.83328pt}|u|^{-1}$
4	$\exp \Bigl [1 - \sqrt{1 + m y / 2}\Bigr ]$	$\frac{2}{m} \, \bigl (L^2(u) + 2 L(u)\bigr )$	0	$\frac{4}{m} \, |u|^{-1} \, \bigl (L(u) + 1\bigr )$

## Interpolation of DPDs in the distance between partons

In the discussion so far, the transverse distance *y* between partons did not play an active role, given that evolution and flavor matching of DPDs are performed for each discretized value of *y* individually, without any cross talk between different values of *y*. Once a DPD is computed for selected scales $(\mu _1, \mu _2)$ and flavor numbers $(n_{1}, n_{2})$, two different tasks are concerned with the *y* dependence:

1. interpolation of the DPD for specific values of *y*, in order to study how evolution and matching have modified the *y* dependence of the initial conditions. In the study described in Ref. [81], visible effects of evolution on the large-*y* dependence of DPDs were reported, and in Ref. [24] it was found that evolution can substantially flatten the $y^{-2}$ behavior generated by the perturbative splitting mechanism at small distances. Numerically reliable interpolation of DPDs over a wide range of *y* is hence important for understanding the dynamics of evolution.
2. integration over *y* from a minimum value $y_{\min }$ up to infinity. First and foremost, this is needed for computing the double parton luminosities (6) that enter in cross sections of double parton scattering processes. It is also needed for evaluating sum rules (see Sect. 7), which DPDs must satisfy and which provide valuable constraints for their modeling. Note that one integrates the product of two DPDs when computing double parton luminosities and single DPDs when evaluating the sum rules.

Both tasks can be carried out using Chebyshev grids, as described in Sect. 3.1. To this end, one first needs to find variable transformations that map the characteristic *y* dependence of DPDs to a dependence in the variable *u* that is well suited for interpolation by high-order polynomials. In the next two subsections, we show how this can be achieved. After that, we will demonstrate the resulting accuracy of interpolation and integration.

### Characteristic *y* dependence and associated variable transformations

It is natural to distinguish the region of small *y*, where perturbative mechanisms are at work from the domain of large *y*.

As mentioned earlier and discussed at length in Ref. [24], DPDs at small *y* can be represented as the superposition of a part due to perturbative splitting, with a power behavior like $y^{-2}$, and an “intrinsic” part that behaves like $y^{0}$ and is related to twist-four distributions. Both power laws are modified by logarithms from loop corrections. The relative weight of the two parts strongly depends on the parton combination and on the momentum fractions $x_1$ and $x_2$ in the DPD. As already mentioned, the $y^{-2}$ behavior can be substantially flattened by evolution to high scales. In the small-*y* region, the transformation *u*(*y*) should therefore be suited to handle the interpolation of a power behavior $y^{-q}$ with $0 \le q \le 2$.

A complication arises from the fact that for certain parton combinations, such as *uu* or $u {\bar{d}}$, the leading-order splitting contribution (7) is zero. Quark-gluon mixing results in nonzero values for these combinations when evolving from the scale $\mu _y \sim 1/y$ at which the splitting formula is evaluated to the final scale $\mu$. Hence, the splitting part of the DPD at scale $\mu$ has a zero around $y \sim 1/\mu$. The position of this zero is shifted when the intrinsic part is added.

At large distances, one expects a gradual decrease of DPDs governed by some characteristic nonperturbative mass scale. This is often modeled by a Gaussian, $F \sim \exp (- M^2 y^2)$, but one may also argue in favor of a Yukawa-like decrease, $F \sim \exp (- M y)$. The characteristic mass scale *M* may depend on the parton combination and on $x_1$ and $x_2$ [41, 81], and it can slowly change under evolution.

We now discuss variable transformations that we found to work well for the types of *y* dependence just discussed. An overview of the transformations and their properties is given in Tables 1 and 2. By convention, all transformations are such that *u*(*y*) has a positive derivative in the domain where it is used. In the case of interpolation, the function *f*(*y*) in Table 2 represents a DPD at fixed momentum fractions or scales. For sum rule integrals *f*(*y*) is $2 \pi y$ times a DPD (see Eq. (55)), and for double parton luminosities *f*(*y*) is $2 \pi y$ times the product of two DPDs (see Eq. (52)). The overall normalization of *f*(*y*) is irrelevant for the interpolation or integration accuracy and therefore set to an arbitrary value here.

**Table 2:** Behavior of the variable transformations in Table 1 for selected functions *f*(*y*). In the third column we used $\exp \bigl [ -c \hspace{0.83328pt}L(u) \bigr ] = |u|^{c}$. The condition in the last column ensures that $\textrm{d}y/ \textrm{d}u \, f\bigl ( y(u) \bigr ) \rightarrow 0$ for $u\rightarrow 0$. It is understood that the parameters *M* and *m* are always positive

No.	*f*(*y*)	$f\bigl ( y(u) \bigr )$	$\int ^\infty \textrm{d}y \, f(y)$ requires
1	$y^{-\beta }$	$|u|^{\hspace{0.83328pt}\beta / \alpha }$	$\alpha < \beta - 1$
2	$\exp (-M^2 y^2)$	$|u|^{\hspace{0.83328pt}(2 M / m)^2} \, \exp \Bigl [ \frac{M^2}{m} \hspace{0.83328pt}y(u) \Bigr ]$	$m < 2 M$
3	$\exp (-M^2 y^2)$	$|u|^{\hspace{0.83328pt}L(u) \; (4 M / m)^2}$	No condition
3	$\exp (-M y)$	$|u|^{\hspace{0.83328pt}4 M /m}$	$m < 4 M$
4	$\exp (-M y)$	$|u|^{\hspace{0.83328pt}L(u) \; 2 M /m} \; |u|^{\hspace{0.83328pt}4 M /m}$	No condition

The last three transformations in the table are designed for functions with a decrease like $\exp (- M^2 y^2)$ or $\exp (- M y)$ at large *y*, possibly modified by a power law in *y*. They map the physical interval $y \in [0, \infty ]$ onto $u \in [-1, 0]$ and depend on a mass parameter *m*, which is normalized such that $\textrm{d}y / \textrm{d}u = 4/m$ at $y=0$. The integral

43 $$\begin{aligned} \int _{y_{\min }}^{\infty } \!\! \textrm{d}y \; f(y) =\int _{u_{\min }}^{0} \!\! \textrm{d}u \; \frac{\textrm{d}y}{\textrm{d}u} \, f\bigl (y(u)\bigr ) \end{aligned}$$

is computed with the Clenshaw–Curtis quadrature rule, which includes the interval boundary $u=0$ where the Jacobian $\textrm{d}y / \textrm{d}u$ is infinite. The upper limit on *m* specified in the last column of Table 2 ensures that the product $\textrm{d}y / \textrm{d}u \, f\bigl (y(u)\bigr )$ is zero at the point $u = 0$, so that this point does not contribute to the Clenshaw–Curtis rule. We note that the value of the mass scale *M* in the integrand depends on whether one integrates a product of two DPDs or a single DPD.

**Inverse power law:** Transformation no. 1 in the table is $u(y) = - y^{-\alpha }$. We will show that with $\alpha \sim 0.2$ this is well suited for the range of power laws $y^{-\beta }$ expected for DPDs at small distances. The more general form $u(y) = - (y + a)^{-\alpha }$ can be used down to $y=0$ if $a>0$, but we will not need this here.
**Gaussian:** Transformation no. 2 in the table has a Gaussian falloff at large *y* and is designed for functions with a Gaussian behavior, $$f(y) \sim \exp (- M^2 $$ $$ y^2)$$. The transformed function $$f\bigl ( y(u) \bigr )$$ approximately behaves like a power of |*u*| for $$u\rightarrow 0$$, and we find that good interpolation and integration accuracy is obtained for $$m \sim M$$.
**Exponential:** Transformation no. 3 in the table is an exponential in *y*. It is suitable for both *f*(*y*) $\sim \exp (- M y)$ and $f(y) \sim \exp (- M^2 y^2)$. In the first case, the transformed function *f* $\bigl ( y(u) \bigr )$ behaves like a power of |*u*| for $u\rightarrow 0$, whereas in the second case it falls off faster than any power of |*u*|. In both cases, values $m \sim M$ yield good interpolation and integration accuracy.
**Exponential with square root:** Transformation no. 4 in the table is designed for functions $f(y) \sim \exp (- M y)$ with a Yukawa-type decrease. The transformed function *f*$\bigl ( y(u) \bigr )$ decreases faster than any power of |*u*| for $u\rightarrow 0$, and good accuracy is obtained with $m \sim M$.

When handling DPDs with different flavor combinations and in different kinematic regions, the mass scale *M* of *f*(*y*) in the preceding discussion does not have a unique value. *M* should instead be understood as an average, or in the case of the bounds in the last column of Table 2 as the lower limit of the relevant values.

### Composite grids

Given the different behavior of DPDs at small and large *y*, it is natural to use interpolation grids with different transformations *u*(*y*). At small *y*, there is an additional physics reason for using several subgrids. Not only the scale $\mu _y \sim 1/y$ but also the number $n_{}$ of active flavors for which the splitting DPD is naturally initialized depends on *y*. Since the value of a DPD in general changes with $n_{}$, one should use a unique value of $n_{}$ in a subgrid, so as to avoid interpolating discontinuous functions. As discussed at length in Ref. [82], it is appropriate to evaluate the perturbative splitting formula (7) with $n_{}$ active flavors for $\gamma \hspace{0.83328pt}m_{n}< \mu _y < \gamma \hspace{0.83328pt}m_{n+1}$ with $\gamma \sim 1$. We therefore place subgrid boundaries in *y* at values around the inverse heavy quark masses.

In the following studies, we will assume a *y* dependence

44 $$\begin{aligned} F(y; \kappa ) = y^{-2 \kappa } \, \exp (-M^2 y^2), \end{aligned}$$

for a single DPD, with $\kappa = 0$ for the intrinsic and $\kappa = 1$ for the splitting part. We take $M = 0.21 \,{\textrm{GeV}}$, which roughly corresponds to the Gaussian parameter in quark-gluon DPDs of the model used in Refs. [24, 30]. As for the function *f*(*y*) in Table 2, the overall normalization of $F(y; \kappa )$ is irrelevant for the interpolation accuracy.

**Fig. 13:**
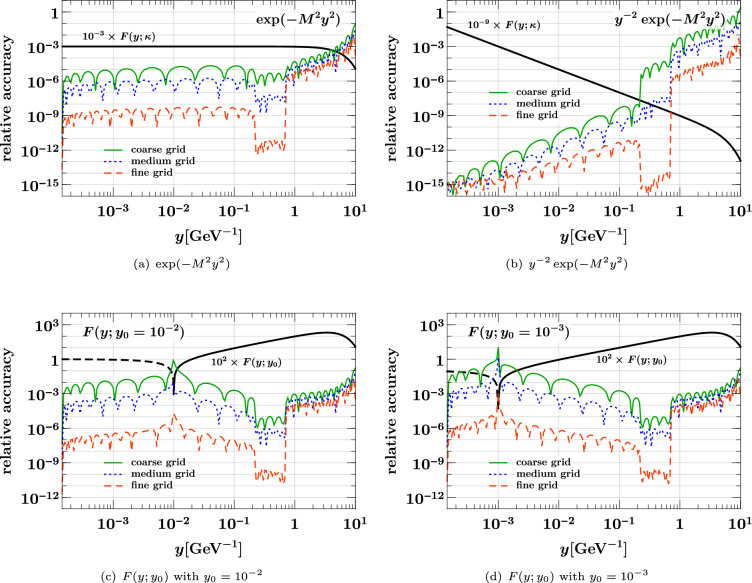
Relative interpolation accuracy (38) on the grids specified in Eqs. (45), (46) and (47). The interpolated functions are $F(y; \kappa )$ in the upper panels and $F(y; y_0)$ in the lower ones, see Eqs. (44) and (48). A dashed black line indicates that the interpolated function is negative. Here and in all following plots, the mass parameter in the functions is $M = 0.21 \,{\textrm{GeV}}$

The smallest value of *y* needed in a given physics setting corresponds to the inverse of the largest hard scale that will be considered. In the following, we will take $y_{\min } = 1 / (7 \,{\textrm{TeV}})$, which covers the production of very heavy states at the LHC. For the interpolation of Eq. (44) and for the computation of the associated double parton luminosities, we use the following combination of grids and transformations:

45 $$\begin{aligned}&\bigl [ \hspace{0.83328pt}1 / (7 \,{\textrm{TeV}}), 1/m_b, 1/m_c \, \bigr ]_{p_1,\, p_2} \nonumber \\&\quad \text {inverse power law transf. with }\alpha = 0.2, \end{aligned}$$

46 $$\begin{aligned}&\bigl [ \hspace{0.83328pt}1/m_c, \infty \, \bigr ]_{p_3}\nonumber \\&\quad \text {Gaussian transf. with }m = 2 M, \end{aligned}$$

where the “inverse power law” and the “Gaussian” transformation are respectively given in entries no. 1 and 2 of Table 1. We find that taking $m = 2 M$ results in a somewhat better overall accuracy than the rule-of-thumb value $m \sim M$ given in the previous subsection. We will consider three different settings for the number of points in each subgrid:

47 $$\begin{aligned} (p_1, p_2, p_3) = {\left\{ \begin{array}{ll} (12, 6, 12) &{} (\text {coarse }y \text { grid)} \\ (16, 8, 16) &{} (\text {medium }y\text { grid)} \\ (24, 12, 24) &{} (\text {fine }y\text { grid)}. \end{array}\right. } \end{aligned}$$

Notice the relatively small number of points in the central grid, which covers the narrow *y* region in which one would initialize the splitting part of the DPD with 4 active flavors. For the quark masses, we use the values specified at the beginning of Sect. 5, which results in subgrid boundaries at $1/m_b \approx 0.22 \,{\textrm{GeV}}^{-1}$ and $1/m_c \approx 0.71 \,{\textrm{GeV}}^{-1}$.

**Fig. 14:**
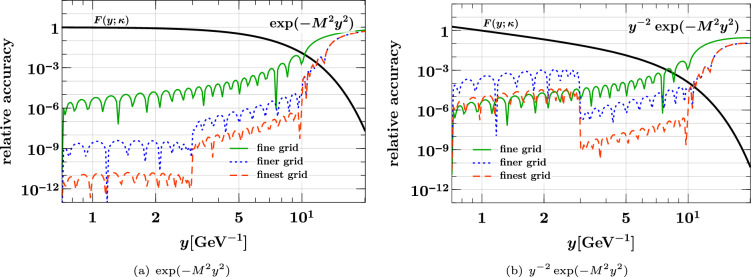
Relative interpolation accuracy at large *y* for the grid in Eq. (46) with $p_3 = 24$ points (fine grid) and for the composite grids specified by Eqs. (49), (50) and (51) (finer and finest grid). The interpolated functions are the same as in Fig. 13a and b, respectively

### Accuracy of interpolation in *y*

In the top panels of Fig. 13, we show the interpolation accuracy for the form (44) with $\kappa = 0$ and $\kappa = 1$. In the first and second subgrid, a very accurate interpolation is obtained even with the coarse grid setting. In the third subgrid, the accuracy degrades rather quickly for large distances, but for the medium grid it remains below 1% for *y* up to about $4 \,{\textrm{GeV}}^{-1} \approx 0.8 {\text {fm}}$. The fine grid yields an acceptable accuracy at even larger distances.

Before discussing this issue in more detail, we consider the interpolation of a DPD with a zero crossing in *y*, which arises for certain parton combinations as explained above. As an example for this case, we take the simple form

48 $$\begin{aligned} F(y; y_0) = (y - y_0) \exp (-M^2 y^2) , \end{aligned}$$

with different values of $y_0$ and the same mass parameter $M = 0.21 \,{\textrm{GeV}}$ as before. The resulting accuracy is shown in the bottom panels of Fig. 13. We find that the coarse grid performs poorly, whereas with the medium grid the relative accuracy stays below 1% except in the immediate vicinity of the zero crossing. Very high accuracy is obtained with the fine grid.

We note that for the intermediate *y* grid from $1/m_b$ to $1/m_c$, the setting with $p_2 = 8$ grid points is sufficient to obtain a relative interpolation accuracy of $10^{-6}$ or better in all cases considered here. In each of the grids for smaller and at larger *y*, one may take either 16 or 24 points, depending on how stringent the accuracy requirements are. This gives 40, 48, or 56 for the total number of points.^4^

With the grids considered so far, the interpolation accuracy at very large *y* becomes poor mainly because there are too few grid points in that region. For many purposes, the behavior of the distributions in the extreme tail region is not of particular interest, and it contributes only little to integrals over *y*. Nevertheless, we wish to show that with an appropriate choice of subgrids and grid transformations, one can also accurately describe this region. To this end, we replace the large-*y* grid in Eq. (46) with

49 $$\begin{aligned}&\bigl [ 1 / m_c, 3 \,{\textrm{GeV}}^{-1}, 10 \,{\textrm{GeV}}^{-1} \bigr ]_{p_{a},\, p_{b}} \nonumber \\&\quad \text {Gaussian transf. with }m = \sqrt{2} M, \end{aligned}$$

50 $$\begin{aligned}&\bigl [ 10 \,{\textrm{GeV}}^{-1}, \infty \bigr ]_{p_{c}}\nonumber \\&\quad \text {Gaussian transf. with }m = 2 M, \end{aligned}$$

where we consider two settings for the number of points:

51 $$\begin{aligned} (p_{a}, p_{b}, p_{c}) = {\left\{ \begin{array}{ll} (12, 12, 8) &{} (\text {finer } y\text { grid)} \\ (16, 16, 8) &{} (\text {finest } y\text { grid}). \end{array}\right. } \end{aligned}$$

In Fig. 14, the resulting interpolation accuracy is compared with the previous setting of Eq. (46) with $p_3 = 24$ points. We see that both the finer and the finest grid extend the domain of very accurate interpolation up to $y \approx 10 \,{\textrm{GeV}}^{-1} \approx 2 {\text {fm}}$, with only a moderate increase in the number of grid points.

**Fig. 15:**
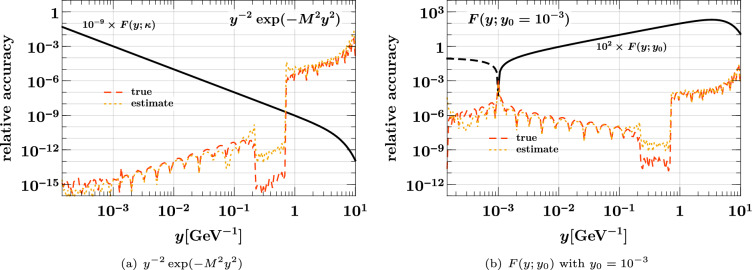
True and estimated relative interpolation accuracy in *y*, as defined in Eqs. (38) and 40, respectively. The curves are for the fine grid setting in Eq. (47)

In analogy to Sect. 4.3, we can estimate the interpolation accuracy in *y* by comparing the results of interpolation with and without subinterval end points. We find that this method works very well, as shown by the examples in Fig. 15. The biggest discrepancies between true and estimated errors appear near subgrid boundaries, and in the central grid, where the omission of the end points decreases the polynomial order of interpolation from 11 to 9.

### Accuracy of integration over *y*

Integration over *y* from a lower cutoff $y_{\min }$ to infinity is needed to evaluate the double parton luminosities (6) and DPD sum rules. If $y_{\min }$ coincides with the end point of a subinterval of the interpolation grid, one can directly use Clenshaw–Curtis quadrature to compute the integral. Otherwise, we perform a number of intermediate steps:

1. identify the subinterval $[y_a, y_b]$ that contains $y_{\min }$,
2. define a reduced subgrid on the interval $[y_{\min }, y_b]$ with the same number of points and the same variable transformation as the original subgrid,
3. compute the required DPDs on the reduced subgrid by interpolation on $[y_a, y_b]$.

The integral is then computed using Clenshaw–Curtis quadrature on the reduced subgrid and on the subgrids for $y \ge y_b$.

Let us investigate the accuracy of this procedure for the double parton luminosities

52 $$\begin{aligned} {\mathcal {L}}(y_{\min }, \kappa _1 + \kappa _2)&= 2 \pi \!\! \int \limits _{y_{\min }}^{\infty } \! \textrm{d}y \; y \, F(y; \kappa _1) \, F(y; \kappa _2)\nonumber \\&= 2 \pi \!\! \int \limits _{y_{\min }}^{\infty } \! \frac{\textrm{d}y\; y}{y^{\hspace{0.83328pt}2 (\kappa _1 + \kappa _2)}} \, \exp (-2 M^2 y^2) \end{aligned}$$

that correspond to the simplified shapes of DPDs in Eq. (44). We consider the cases $\kappa _1 + \kappa _2 = 0, 1, 2$, which respectively correspond to taking the intrinsic parts of both DPDs, the intrinsic part of one and the splitting part of the other DPD, or the splitting parts of both DPDs. Although only the sum of intrinsic and splitting parts enters in physical cross sections, it is useful to investigate these different combinations separately, as was for instance done in the studies in Refs. [24, 82].

**Fig. 16:**
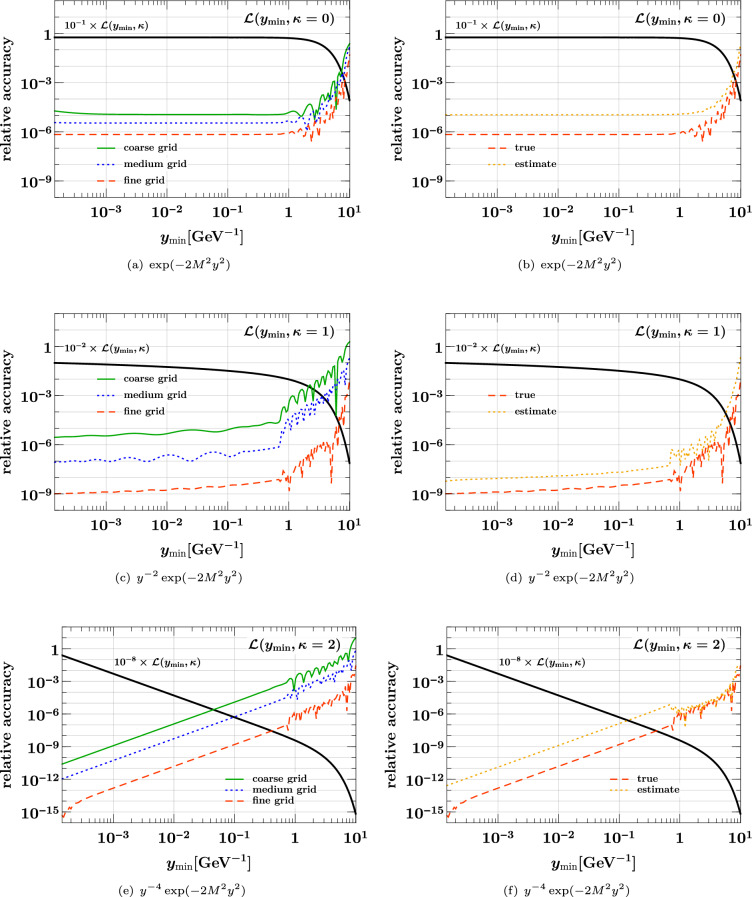
Left: Relative accuracy of computing the integrals (52) with the interpolation grids specified by Eqs. (45), (46) and (47). Right: True and estimated integration accuracy for the fine grid setting of Eq. (47). The functions in the legends are the product $F(y; \kappa _1)\, F(y; \kappa _2)$ of DPDs in the luminosity integral

The integration accuracy can be computed using the exact expression of (52) in terms of an incomplete gamma function. The result is shown in the left panels of Fig. 16 for the interpolation grids introduced in Sect. 6.2. We see that for $y_{\min }$ below $1 \,{\textrm{GeV}}^{-1}$, good accuracy is achieved even with the coarse grid. For cutoff values above $1 \,{\textrm{GeV}}^{-1}$, the accuracy quickly becomes unsatisfactory unless the fine grid is used. This reflects the degrading interpolation accuracy in that region, which was shown in the upper panels of Fig. 13. In physics applications, $y_{\min }$ is of the order of an inverse hard scale, so that large values of the cutoff will only be needed in special circumstances, for instance if one is interested in the partial contribution of large *y* to an integral. We see that the fine grid setting is useful in such a case.

In the right panels of Fig. 16, we compare the true integration accuracy with its estimate obtained from comparing the values obtained with Clenshaw–Curtis quadrature and with Fejér’s second rule (see Sect. 3.1). We see that for $y_{\min }$ below $1 \,{\textrm{GeV}}^{-1}$, one obtains an overestimate of the true error by one or two orders of magnitude. This is qualitatively similar to what we found for integrals of PDFs over *x* in section 3.4 of Ref. [55].

## Cross check: comparison with DOVE

**Fig. 17:**
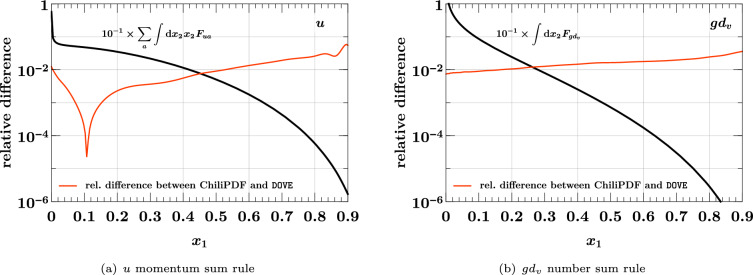
Relative differences between the left-hand sides of the DPD sum rules (53) and (54), evaluated using DOVE and ChiliPDF. Details are given in the text

As a cross check for our implementation of DPD evolution in ChiliPDF, we performed a comparison with DOVE, a code that was introduced in Ref. [28] and further developed or adapted in subsequent work in Refs. [24, 30, 44, 45, 81].^5^ The comparison is done with the version that was used in Ref. [30] to compute the Gaunt-Stirling sum rules for DPDs. In the following, we show the momentum sum rule for a *u* quark and the number sum rule for the parton combination $g d_v$, which read53and54where $F_{g \hspace{0.83328pt}\smash {d_v}}^{} = F_{g \smash {d}}^{} - F_{g \smash {{\bar{d}}}}^{}$. The expressions for general parton combinations are given in Eqs. (3.1) and (3.2) of Ref. [28]. As shown in Ref. [83], the sum rules hold for the distributions55which are integrated over *y* with an $\overline{\text {MS}}$ renormalization prescription for the $y^{-2}$ singularity due to perturbative splitting. The matching term $F_{a_1 a_2}^{\text {match}}(x_1, x_2; \nu , \mu )$ relates this prescription with the cutoff in *y* used on the r.h.s. of Eq. (55). It can be computed in terms of a PDF and a matching kernel in a similar fashion as the splitting part in Eq. (7), and its LO expression is given in Eq. 18 of Ref. [30]. The dependence on the matching scale $\nu$ cancels between the two terms in (55) up to power corrections in $1/\nu ^2$. We recall that $b_0 = 2 e^{-\gamma _E} \approx 1.12$.

The different roles played by $x_1$ and $x_2$ in the sum rules (53) and (54) motivate us to take different grids for the two momentum fractions,

56 $$\begin{aligned} x_1:&\quad \bigl [ 5 \times 10^{-5}, 5 \times 10^{-3}, 0.5, 0.9, 1.0 \bigr ]_{(12, 12, 16, 8)} , \nonumber \\ x_2:&\quad \bigl [ 5 \times 10^{-5}, 5 \times 10^{-3}, 0.5, 1.0 \bigr ]_{(12, 16, 16)} . \end{aligned}$$

The first grid has an additional small subgrid that leads to a better interpolation accuracy at large $x_1$, which is not needed for integration over $x_2$. The sum rule integrals over $x_2$ are computed using Clenshaw–Curtis quadrature over the full $x_2$ grid. This implies that the integrals are computed with a lower cutoff $x_{\min } = 5 \times 10^{-5}$. The same cutoff is used in the computation with DOVE.

We find that the integration over *y* in the sum rules can be computed with the rather small grids

57 $$\begin{aligned}&\bigl [ b_0 / (170 \,{\textrm{GeV}}), b_0 / (1 \,{\textrm{GeV}}) \bigr ]_{(12)} \nonumber \\&\quad \hbox { inverse power law transf. with}\ \alpha = 0.2 , \end{aligned}$$

58 $$\begin{aligned}&\bigl [ b_0 / (1 \,{\textrm{GeV}}), \infty \bigr ]_{(8)} \nonumber \\&\quad \hbox { Gaussian transf. with}\ m = M , \end{aligned}$$

where $M = 1 \big / \sqrt{4 h_{g g}} \approx 0.23 \,{\textrm{GeV}}$ is the Gaussian mass parameter for gluons in the initial conditions of the model (see Eq. (8)).

We take the DPD model specified in section 3 of Ref. [30] and evolve to $\mu _1 = \mu _2 = 144.6 \,{\textrm{GeV}}$ with $n_{} = 3$ active flavors for each parton. At that scale, the momentum and number sum rules are evaluated with $\nu = 144.6 \,{\textrm{GeV}}$. These $\mu$ and $\nu$ values correspond to grid points in our setup of DOVE and thus avoid additional interpolation. The detailed grid settings and integration procedure in DOVE are as specified in section 4 of Ref. [30].

In Fig. 17, we show the relative deviation between the left-hand sides of the sum rules (53) and (54) evaluated in ChiliPDF and in DOVE. The numerical differences increase with $x_1$ and reach several percent at $x_1 = 0.9$. For sum rules with other parton combinations, we find similar or smaller discrepancies. We also compared the sum rules evaluated at smaller values of $\mu _1 = \mu _2$ and $\nu$ and found somewhat smaller discrepancies than at high scales.

An error estimate for the accuracy of evolution with DOVE in its original version is given in figure 9 of [28]. Given that this estimate was carried out with finer grids than the ones used here, and given that relatively simple integration rules are used in the evaluation of the sum rules with DOVE, we find the level of agreement between the two codes satisfactory for the purpose of cross-validation.

## Conclusions

We have shown that discretization on Chebyshev grids allows for a precise and economical representation of DPDs as functions of the momentum fractions $x_1$, $x_2$ and the distance *y* between the partons. This generalizes the methods we developed for PDFs in Ref. [55]. We use grids for $x_1$ that are independent of $x_2$ and vice versa. This gives the highest degree of simplicity and decoupling between operations that involve only one momentum fraction, which is the case for the Mellin convolutions that appear in DGLAP evolution and in flavor matching. This method incurs interpolation errors close to the kinematic boundary $x_1 + x_2 = 1$ of the DPDs, which we find manageable. We investigated the interpolation accuracy for typical shapes of DPDs, both for the input distributions and after evolution. We find that grids with $p_{x} = 54$ points for $x \ge 10^{-5}$ yield a very accurate representation of DPDs, with relative errors that are much smaller than $10^{-3}$ in large parts of the phase space and remain below $1\%$ for $x_1 + x_2 < 0.8$ (see Figs. 10 and 11).

For the dependence on *y*, Chebyshev interpolation is used after variable transformations that are adapted to the functional form and the typical mass scale for the decrease of the DPDs at large distance. We find that with $p_{y} \sim 48$ grid points, very good accuracy can be achieved for interpolation and integration.

To solve the DGLAP equations for DPDs, we use a high-order Runge–Kutta algorithm for computing the evolution matrices in Eqs. (33) and (34), which are the discretized version of the Green functions for the evolution equations. This provides very high numerical accuracy for both forward and backward evolution, with errors well below the ones due to discretization in $x_1$ and $x_2$. With our algorithm, it is natural to evolve DPDs separately in the scales $\mu _1$ and $\mu _2$ associated with the two partons. We note that the computational effort scales linearly with $p_{y}$ but with the third power of $p_{x}$.

In our setup, a DPD with given flavor numbers $(n_{1}, n_{2})$ is specified numerically by its values at all grid points in $(x_1, x_2, y)$ and at one pair $(\mu _1, \mu _2)$ of scales. Its values at other scales are computed “on the fly” by solving the evolution equations. DPDs at other values of $x_1$, $x_2$, and *y* are computed using the barycentric interpolation formula, which is fast and numerically stable. As discussed in the introduction, we avoid grids in the renormalization scales, which would require a huge amount of computer memory. This is a notable difference between our approach and the widely used method to compute PDFs from grids in *x* and $\mu$, for instance via the LHAPDF interface [54], and it reflects the notable difference in complexity between the distribution functions for one or for two partons inside a proton.

With our present implementation of the above methods in ChiliPDF, the evolution of a DPD with $n_{} = 5$ active flavors (and thus 121 parton flavor combinations) from $\mu _1 = \mu _2 = 15 \,{\textrm{GeV}}$ to $150 \,{\textrm{GeV}}$ takes 1 to 2 s^6^ for $p_{x} = 54$ grid points in $x_1$ and $x_2$ and $p_{y} = 48$ grid points in *y*. With grids of this size, the largest part of computing time is spent on multiplying the evolution matrices with the initial conditions, so that there is only a weak dependence of the timing on the perturbative order of evolution and on the initial and final scales. Since the current code is based on a simple implementation of linear algebra and matrix multiplication, we expect that significant gains can still be made with a dedicated performance tuning.

We used our code to investigate the impact of higher orders in the DGLAP kernels on the evolution of DPDs. For some parton combinations, the change from LO to NLO evolution produces ${\mathcal {O}}(1)$ effects at small momentum fractions, whereas we find only moderate differences when changing from NLO to NNLO kernels.

The numerical delivery of realistic DPDs has been a bottleneck in practice so far. The presented methods and their implementation provide a practical tool for computing evolved DPDs from user-given starting conditions, on a par with what has long been a standard for PDFs. This increases our ability to use the predictive power of perturbation theory and thus presents an important ingredient to making theory predictions for double parton scattering more realistic.

The evolution equations considered in this work apply to DPDs that are summed separately over the color of each parton. DPDs with color correlations between the two partons follow a different evolution pattern, with kernels that have recently been computed at NLO [84]. The methods described in the present paper can be adapted to this case, as will be reported in a future publication.

## Data Availability

This manuscript has no associated data or the data will not be deposited. [Authors’ comment: There is no associated data for this paper.]

## Appendix A: Path independence of evolution and flavor matching

In this appendix we show that the set (1) of DGLAP equations has a unique solution for a given initial condition at a point in the $(\mu _1, \mu _2)$ plane. In other words, the solution is independent of the path in the $(\mu _1, \mu _2)$ plane that is taken to evolve from the initial condition to the final scales. To establish this, it is enough to show that the second derivatives in the renormalisation scales commute, i.e. that

59 $$\begin{aligned}&\frac{\partial }{\partial L_1} \, \frac{\partial }{\partial L_2} \, F^{n_{1}, n_{2}}_{a_1 a_2}(L_1, L_2) \nonumber \\&\quad \! = \frac{\partial }{\partial L_1} \biggl ( \sum _{b_2} P^{n_{2}}_{a_2 b_2}(L_2) \underset{2}{\otimes } F^{n_{1}, n_{2}}_{a_1 b_2}(L_1, L_2) \biggr ) \nonumber \\&\quad \! = \sum _{b_2, b_1} P^{n_{2}}_{a_2 b_2}(L_2) \underset{2}{\otimes } P^{n_{1}}_{a_1 b_1}(L_1) \underset{1}{\otimes } F^{n_{1}, n_{2}}_{b_1 b_2}(L_1, L_2) \end{aligned}$$

is equal to

60 $$\begin{aligned}&\frac{\partial }{\partial L_2} \, \frac{\partial }{\partial L_1} \, F^{n_{1}, n_{2}}_{a_1 a_2}(L_1, L_2) \nonumber \\&\quad \! = \frac{\partial }{\partial L_2} \biggl ( \sum _{b_1} P^{n_{1}}_{a_1 b_1}(L_1) \underset{1}{\otimes } F^{n_{1}, n_{2}}_{b_1 a_2}(L_1, L_2) \biggr ) \nonumber \\&\quad \! = \sum _{b_1, b_2} P^{n_{1}}_{a_1 b_1}(L_1) \underset{1}{\otimes } P^{n_{2}}_{a_2 b_2}(L_2) \underset{2}{\otimes } F^{n_{1}, n_{2}}_{b_1 b_2}(L_1, L_2) . \end{aligned}$$

Here and in the following, we change evolution variables from $\mu _i$ to

61 $$\begin{aligned} L_i = 2 \ln \mu _i \end{aligned}$$

and drop the arguments $x_1, x_2$, and *y* of the DPDs for brevity. For evolution at fixed $(n_{1}, n_{2})$, i.e. without flavor matching, the desired equality follows from the fact that the convolution integrals w.r.t. the first and second momentum fraction of the DPD commute with each other. This can readily be shown from their definition (2). Note that it is essential for the argument that the evolution kernel for one parton does not depend on the scale of the other parton.

We now extend this argument, showing that one obtains the same result when flavor matching along different paths in the $(n_{1}, n_{2})$ plane. Let us see how Eqs. (59) and (60) have to be modified to include the effects from flavor matching. We first consider the case of PDFs, where the notation is less involved. For a set of given matching scales ${\tilde{\mu }}_{n}$ for the transition from $n - 1$ to *n* active flavors, we define the “canonical” number of flavors at given $L = 2 \ln \mu$ as

62 $$\begin{aligned} n(L)&= {\left\{ \begin{array}{ll} 3 &{} \hbox {for}\ L< {\widetilde{L}}_{4} \\ 4 &{} \hbox {for}\ {\widetilde{L}}_{4}< L< {\widetilde{L}}_{5} \\ 5 &{} \hbox {for}\ {\widetilde{L}}_{5}< L < {\widetilde{L}}_{6} \\ 6 &{} \hbox {for}\ L > {\widetilde{L}}_{6} \end{array}\right. } \end{aligned}$$

where ${\widetilde{L}}_{n} = 2 \ln {\tilde{\mu }}_{n}$. We further define PDFs

63 $$\begin{aligned} f^{}_{a}(L) = f_{a}^{n(L)}(L) , \end{aligned}$$

with the canonical number of flavors at a given renormalisation scale. From these distributions, one can obtain $f^{n}_{a}(L)$ for flavor numbers different from the canonical values by evolution.^7^

At the matching scales, the distributions in Eq. (63) can have discontinuities, which complicates their description via differential equations. We therefore introduce a smoothened version of $f^{}_{a}(L)$. Taking a single matching step from $n-1$ to *n* flavors at ${\widetilde{L}} = 2 \ln {\tilde{\mu }}$ for simplicity, one can define a smooth approximation $\theta _{\epsilon }(L)$ of the step function that takes the values

64 $$\begin{aligned} \theta _{\epsilon }(t) = {\left\{ \begin{array}{ll} 0 &{} \hbox {for}\ t < - \epsilon \\ 1 &{} \hbox {for}\ t > \epsilon \end{array}\right. } \end{aligned}$$

and is continuous and monotonic in the intermediate region $- \epsilon< t < \epsilon$. Its derivative $\delta _{\epsilon }(t) = \textrm{d}\theta _{\epsilon }(t) / \textrm{d}t$ is then a smooth approximation of the Dirac distribution. Define now a PDF $f^{\epsilon }_{a}(L)$ that evolves according to

65 $$\begin{aligned}&\frac{\textrm{d}f^{\epsilon }_{a}(L)}{\textrm{d}L} = \sum _{b} P_{a b}^{n_{\epsilon }(L)}(L) \underset{}{\otimes } f^{\epsilon }_{b}(L) \nonumber \\&\quad + \delta _{\epsilon }(L - {\widetilde{L}}) \biggl [ \sum _{b} A_{a b}^{n}(m; {\widetilde{L}}) \underset{}{\otimes } f^{\epsilon }_{b}({\widetilde{L}} - \epsilon )- f^{\epsilon }_{a}({\widetilde{L}} - \epsilon ) \biggr ] , \end{aligned}$$

with

66 $$\begin{aligned} n_{\epsilon }(L) = n - 1 + \theta _{\epsilon }(L - {\widetilde{L}}) \end{aligned}$$

interpolating smoothly between $n-1$ and *n*. The DGLAP kernels for the evolution of $f^{\epsilon }_{a}(L)$ are thus evaluated with flavor number $n-1$ for $L \le {\widetilde{L}} - \epsilon$, with flavor number *n* for $L \ge {\widetilde{L}} + \epsilon$, and with fractional flavor number in the intermediate *L* region.^8^ One furthermore has

67 $$\begin{aligned}&f^{\epsilon }_{a}({\widetilde{L}} + \epsilon ) = f^{\epsilon }_{a}({\widetilde{L}} - \epsilon ) + \int _{{\widetilde{L}} - \epsilon }^{{\widetilde{L}} + \epsilon } \textrm{d}L \, \frac{\textrm{d}f^{\epsilon }_{a}(L)}{\textrm{d}L}\nonumber \\&\quad = f^{\epsilon }_{a}({\widetilde{L}} - \epsilon ) + {\mathcal {O}}(\epsilon ) + \int _{{\widetilde{L}} - \epsilon }^{{\widetilde{L}} + \epsilon } \textrm{d}L \; \delta _{\epsilon }(L - {\widetilde{L}}) \nonumber \\&\qquad \times \biggl [ \sum _{b} A_{a b}^{n}(m; {\widetilde{L}}) \underset{}{\otimes } f^{\epsilon }_{b}({\widetilde{L}} - \epsilon ) - f^{\epsilon }_{a}({\widetilde{L}} - \epsilon ) \biggr ] \nonumber \\&\quad = \sum _{b} A_{a b}^{n}(m; {\widetilde{L}}) \underset{}{\otimes } f^{\epsilon }_{b}({\widetilde{L}} - \epsilon ) + {\mathcal {O}}(\epsilon ) , \end{aligned}$$

where the ${\mathcal {O}}(\epsilon )$ contribution comes from the integral over the term with $P^{n_{\epsilon }(L)}$ in Eq. (65). In the limit $\epsilon \rightarrow 0$, this tends to the flavor matching prescription given in Eq. (5).

It is straightforward to extend this construction from one to several matching steps and from PDFs to DPDs. In analogy to Eq. (63), DPDs with canonical flavor numbers are defined as

68 $$\begin{aligned} F^{}_{a_1 a_2}(L_1, L_2) = F_{a_1 a_2}^{n(L_1),\, n(L_2)}(L_1, L_2) , \end{aligned}$$

and their smoothened version $F^{\epsilon }_{a_1 a_2}(L_1, L_2)$ evolves according to

69 $$\begin{aligned}&\frac{\partial }{\partial L_1} \, F^{\epsilon }_{a_1 a_2}(L_1, L_2) \nonumber \\&\quad = \sum _{b_1} P^{n_{\epsilon }(L_1)}_{a_1 b_1}(L_1) \underset{1}{\otimes } F^{\epsilon }_{b_1 a_2}(L_1, L_2) \nonumber \\&\qquad + \sum _{n} \delta _{\epsilon }(L_1 - {\widetilde{L}}_{n}) \sum _{b_1} \Delta A^{n}_{a_1 b_1} \underset{1}{\otimes } F^{\epsilon }_{b_1 a_2}({\widetilde{L}}_{n} - \epsilon , L_2) , \nonumber \\&\frac{\partial }{\partial L_2} \, F^{\epsilon }_{a_1 a_2}(L_1, L_2) \nonumber \\&\quad = \sum _{b_2} P^{n_{\epsilon }(L_2)}_{a_2 b_2}(L_2) \underset{2}{\otimes } F^{\epsilon }_{a_1 b_2}(L_1, L_2) \nonumber \\&\qquad + \sum _{n} \delta _{\epsilon }(L_2 - {\widetilde{L}}_{n}) \sum _{b_2} \Delta A^{n}_{a_2 b_2} \underset{2}{\otimes } F^{\epsilon }_{a_1 b_2}(L_1, {\widetilde{L}}_{n} - \epsilon ) , \end{aligned}$$

where we abbreviated

70 $$\begin{aligned} \Delta A^{n}_{a b}(z) = A^{n}_{a b}(z, m_{n}; {\widetilde{L}}_{n}) - \delta _{a b} \, \delta (1-z) . \end{aligned}$$

As at the beginning of this section, one can then show that

71 $$\begin{aligned}&\frac{\partial }{\partial L_1} \, \frac{\partial }{\partial L_2} \, F^{\epsilon }_{a_1 a_2}(L_1, L_2)\nonumber \\&\quad = \frac{\partial }{\partial L_2} \, \frac{\partial }{\partial L_1} \, F^{\epsilon }_{a_1 a_2}(L_1, L_2) , \end{aligned}$$

so that with an initial condition given in the $(L_1, L_2)$ plane, Eq. (69) has a unique solution independent of the evolution path. Taking the limit $\epsilon \rightarrow 0$, the desired result is obtained.

## Footnotes

1 Chebyshev Interpolation Library for PDFs.

2 We use the central PDF from the MSHT20nnlo_as118 set obtained via LHAPDF [54]. The ChiliPDF library includes an interface for importing data from LHAPDF.

3 The first chapters of this book are available on https://people.maths.ox.ac.uk/trefethen/ATAP.

4 Here we count the common end points of neighboring subgrids twice, since different subgrids may contain DPDs with different flavor numbers.

5 Results obtained with an early version of DOVE are given on the website https://gsdpdf.hepforge.org.

6 Timings were obtained on different computers with Intel® Core™ i5, Intel® Core™ i7, or AMD EPYC™ 7513 32-Core processors.
